# Polyembryonic or Apomictic Seeds Enable Fig Trees to Establish New Populations Without Their Pollinating Wasps, a Case Observation in *Ficus gasparriniana*

**DOI:** 10.1002/ece3.72316

**Published:** 2025-10-16

**Authors:** Jianhang Zhang, Li Mei, Xiaomei Wang, Shuai Liao, Hongqing Li, Zhen Zhang

**Affiliations:** ^1^ School of Life and Environmental Sciences Shaoxing University Shaoxing Zhejiang China; ^2^ School of Life Sciences East China Normal University Shanghai China; ^3^ Key Laboratory of Wetland Biodiversity of the Jianhu Basin of Shaoxing Shaoxing University Shaoxing Zhejiang China; ^4^ State Key Laboratory of Plant Diversity and Specialty Crops, South China Botanical Garden Chinese Academy of Sciences Guangzhou Guangdong China; ^5^ South China National Botanical Garden Guangzhou Guangdong China; ^6^ Eastern China Conservation Centre for Wild Endangered Plant Resources Shanghai Chenshan Botanical Garden Shanghai China

**Keywords:** adventitious embryo, fig–wasp coevolution, molecular marker identification, polyploidy, simple sequence repeat

## Abstract

Fig–wasp coevolution has been extensively studied as a fascinating case of extreme plant–insect codiversification, yet little is known about how fig trees reproduce without pollinating fig wasps. This study provides direct evidence that fig trees sustain their populations by producing polyploid and polyembryonic seeds through apomixis without fig wasp pollination. We report herein that the seeds of unpollinated *Ficus gasparriniana* are derived from adventitious embryos formed in the nucellar tissues and show sporophytic apomixis. Apomixis is an important reproductive mode of *F. gasparriniana*, which has diverse pedigree sources. Flow cytometry combined with chromosome counting and short tandem repeat typing results showed that apomixis in *F. gasparriniana* was closely related to polyploidy, suggesting that sexual reproduction occurred at the diploid level and apomixis occurred at the polyploid level. Thus, other polyploid *Ficus* species may also exhibit apomictic reproduction. This study provides essential data for advancing research on apomixis in *Ficus* and its role in the coevolution of fig–wasp mutualism.

## Graphical abstract

Development of apomictic adventitious embry o in *Ficus gasparriniana*. Apomixis is a key reproductive strategy for *F. gasparriniana*, especially when pollination by fig wasps is limited. In the absence of pollination, *F. gasparriniana* produces seeds through adventitious embryos formed within the nucellar tissue, which is accompanied by polyploidization. The number of seeds produced by apomixis is sufficient to meet the reproductive needs of *F. gasparriniana*. This study provides the first case for understanding apomictic reproduction in *Ficus*.

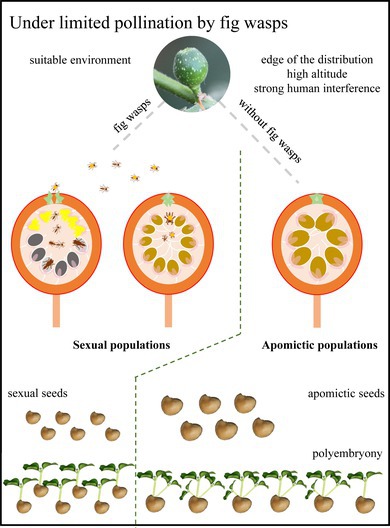

## 1 Introduction

*Ficus* is one of the largest genera of woody plants in angiosperms (Cottee‐Jones et al. 2016), widely distributed in tropical and subtropical regions. Half of the species of the genus are monoecious (male, gall, and female flowers in the same syconium, a distinctive closed inflorescence), while the other half of the species are gynodioecious (male and female flowers in separate syconia of different individuals). Their diploid chromosome number is usually 2*n* = 26 (Ohri and Khoshoo 1987). With over 800 species, *Ficus* plays crucial roles in maintaining the ecological balance of pantropical forest ecosystems (Berg and Wiebes 1992; Shanahan et al. 2001; Berg and Corner 2005; Harrison 2005). In addition, fig trees are closely associated with human activities, offering nutritional, environmental, economic, medicinal, and cultural benefits (Kislev et al. 2006; Lev‐Yadun et al. 2006; Tiwari et al. 2015; Shi et al. 2018; Luo et al. 2019; Zhang et al. 2020).

Fig trees are readily identifiable by their syconia. Only specific fig wasps of the family Agaonidae can pollinate the corresponding fig trees in nature, with a coevolution history dating back to more than 60 million years ago (Xu et al. 2011; Cruaud et al. 2012; Zhang et al. 2019). The distinctive syconia of fig trees are unique inverted receptacles with diverse volatile organic compounds and specific morphological and physiological characteristics, allowing them to form a relationship with their corresponding symbiotic species of fig wasps (Ramirez 1974; Wiebes 1979; Berg 1989; Chen et al. 2002, 2010; Yu et al. 2006; Xu and Yang 2008; Yu and Compton 2012; Zhang et al. 2020). The relationship is so close that the extinction of one would inevitably lead to the extinction of the other (Chen et al. 1996). This obligate mutualism is a fascinating example of extreme plant–insect codiversification (Zhang et al. 2020). Despite its stability, this mutualistic pollination system is also fragile because of the unique life history of fig wasps. On disruption of the interdependence, pollination cannot be completed, leading to ecological imbalance (Kjellberg et al. 1987; Jevanandam et al. 2013; Chen et al. 2018). When fig trees are introduced to a new allopatric habitat, such as “islands,” without their pollinators, their reproduction becomes challenging.

Given the vulnerability of this highly specialized mutualism in novel environments, researchers have explored alternative reproductive strategies in *Ficus*, such as apomixis. Reports on apomixis in *Ficus* mainly involve apomixis induced under artificial conditions. During fig breeding programs, researchers at the Nikitsky Botanical Garden in Crimea found that pollinating *Ficus carica* and *F. afghanistanica* with pollen from distantly related Moraceae genera (e.g., *Morus alba* or *Broussonetia* sp.) induced apomixis, and the seedlings cultivated from unfused reproductive seeds did not possess any characteristics of the pollen parent (Arendt 1960). Cytological analysis of the progeny of *F. afghanistanica* produced by pollination with *M. alba* and under the action of penicillin showed that all the seedlings obtained from the seeds had 2*n* = 39 chromosomes (triploid cytotypes), the same number as the initial maternal individual (Zdruikovskaya‐Rikhter 1970; Falistocco 2016). Based on the materials provided by N. K. Arendt and through embryology analysis, it was shown that in the flower clusters of the Kadota variety of *F. afghanistanica*, the volume of the egg cells in the embryo sac significantly increased after being pollinated with mulberry pollen. Additionally, two other examples showed that the integument cells protruded into the embryo sac cavity occupied by the endosperm. Thus, it was speculated that the embryo‐like structure developed from integument cells (Zamotailov 1955). Subsequent studies have further confirmed that when fig flowers are stimulated by external pollen or various inducers, they produce seeds through asexual reproduction. The main source of embryonic development in this asexual reproduction is the ovary cells (Zdruikovskaya‐Rikhter 1970; Arendt and Kazas 1980). The ovule cells may also directly produce adventitious embryos through integumentary somatic cells, such as in *F. roxburghii*, where adventitious embryos have been observed (Winkler 1906; Naumova 2018). In some unpollinated commercial figs, monoecious fruiting (seedless reproductive) has also been observed (Ma et al. 1997). Previous research on apomixis in *Ficus* has been limited to artificially induced cases accidentally identified during fig breeding, with no in‐depth investigation into its causative factors. Moreover, no other reports exist on apomixis in this genus, and its occurrence under natural conditions remains unknown.

**FIGURE 1:**
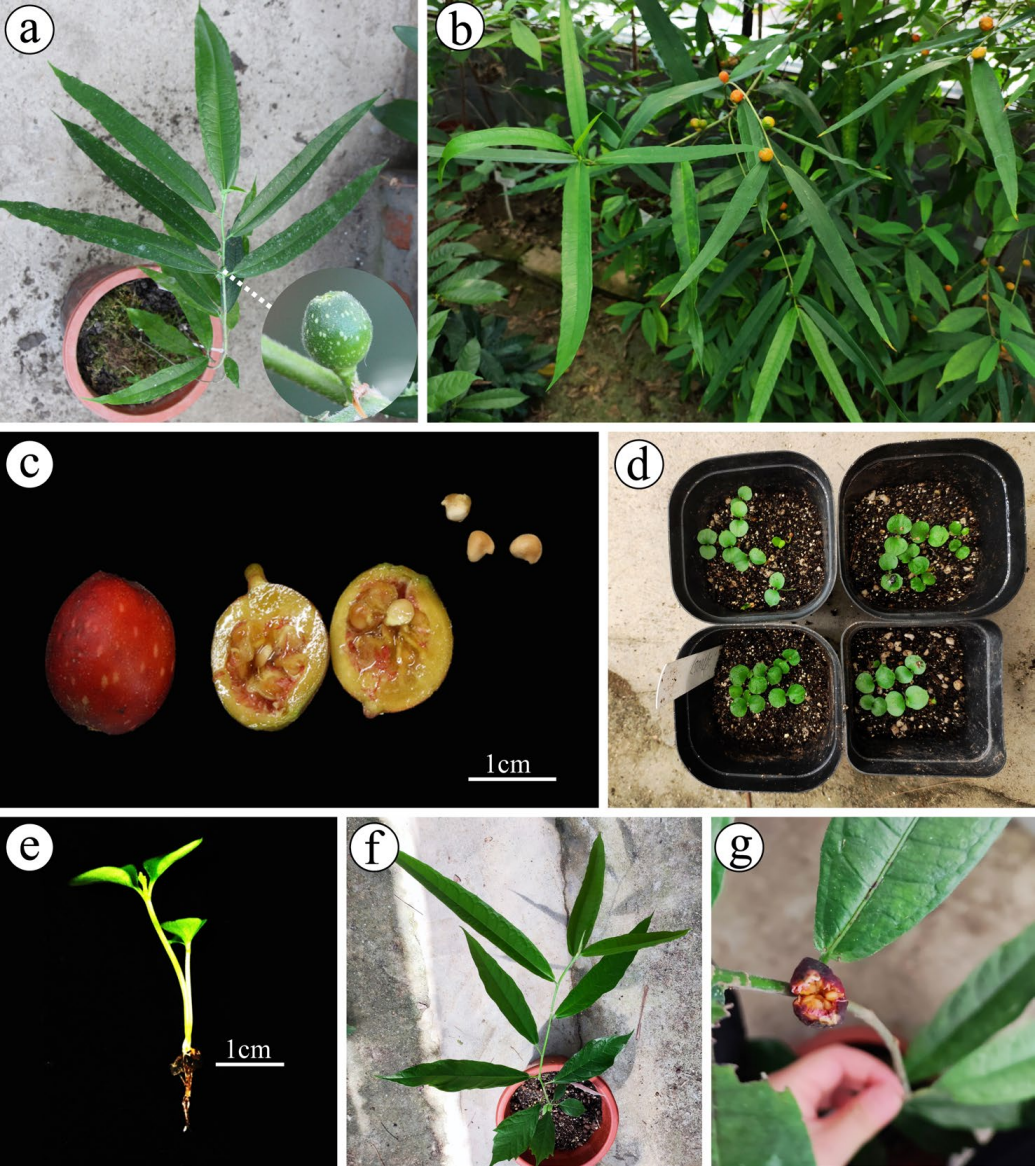
Autonomous cropping of *Ficus gasparriniana* in the greenhouse without fig wasps. (a) Young plant introduced from the wild; (b) mature plants in the greenhouse; (c) mature syconia and drupes (including seeds) from b; (d) seedlings from c; (e) two seedlings from one seed; (f) young plant of the offspring; (g) mature plant from f, producing fertile seeds.

*Ficus gasparriniana*, a dioecious shrub of *Ficus* subgen. *Ficus* (Figure 1), is distributed in southern China, Bhutan, northeastern India, Laos, Myanmar, Thailand, and Vietnam (Zhou and Gilbert 2003). This species relies on fig wasps for pollination and sexual reproduction, and typically does not reproduce vegetatively in natural habitats. However, seedlings of this species, introduced from the wild from Libo County, Guizhou Province, China, produced seeds after 3 years of cultivation in a greenhouse in Shanghai, despite the absence of male flowers and potential pollinators of *F. gasparriniana* and closely related species (Figure 1a–c). These seeds germinated normally (Figure 1d–f), and their seedlings produced fertile seeds again after 2 years (Figure 1g). The morphological characteristics of the new seedlings and their female parent are almost identical (Figure 1a,f). Furthermore, polyembryony (Figure 1e; Figure S1), commonly associated with apomictic reproduction in angiosperms, was observed (Carman 1997; Ozias‐Akins 2006; Echenique et al. 2021). Thus, *F. gasparriniana* may reproduce apomictically in the absence of symbiotic pollinating fig wasps. The phenomenon of *F. gasparriniana* bearing seeds in the absence of its specific pollinating fig wasps raises the following considerations: (1) whether *F. gasparriniana* undergoes apomictic reproduction and of which type; (2) if fruiting in the absence of specific pollinators is common in *F. gasparriniana*, how does this species reproduce in the wild; (3) what is the ploidy level of *F. gasparriniana*, and whether it is an important factor influencing apomictic reproduction; (4) what is the role of apomictic reproduction in the symbiosis system of *F. gasparriniana* and its pollinating fig wasps. These and other issues are explored below to facilitate our understanding of the coevolutionary relationship between figs and their pollinating wasps.

## 2 Materials and Methods

### 2.1 Observation of the Formation and Development of Apomictic Seeds of *F. gasparriniana*

#### 2.1.1 Paraffin Sectioning

Plant materials were obtained from adult *F. gasparriniana* at the Biological Experiment Station of East China Normal University (31°2′11.16″ N, 121°26′55.19″ E). The natural distribution range of *F. gasparriniana* lies over 500 km from the experimental site. The nearest known population within the *Ficus erecta* complex is located on Dajinshan Island, approximately 50 km away. The pollinator of *F. erecta* is highly unlikely to traverse this marine barrier. In the experimental area, only *Ficus pumila* has potential pollinating fig wasps, but they are distantly related and large in size, making it difficult for them to pollinate *F. gasparriniana*. Furthermore, apomictic individuals of greenhouse‐cultivated *F. gasparriniana* provided an additional isolation measure. Considering all these factors, there was no possibility of pollination by fig wasps, so bagging was not carried out. Living materials were collected in 2017 from Libo County, Guizhou Province, China. Fruits of all greenhouse‐introduced *Ficus* plants were removed before plantation. Greenhouse management included regular pesticide application and long‐term placement of sticky insect boards to ensure that no pollinating agents were present. The sampling strategy covered the three important stages of syconium development: female flower development (stage A; Figure S2a,d,g); female flower opening (stage B; Figure S2b,e,h); and syconium growth (increase in volume), seed development, and seed maturation (stage E; Figure S2c,f,i) (Galil and Eisikowitch 1968; Li, Chen, et al. 1999; Li, Zhang, et al. 1999). Several syconia at these stages were collected from three different branches of three trees. Each syconium was longitudinally sectioned to make it easier for soaking paraffin, fixation, and observation. After fixation with formalin–alcohol–acetic acid, female flower development was observed under a stereoscope (Zeiss, Oberkochen, Germany). A total of 18 syconia (36 experimental materials) were vacuum‐packed and stored in a refrigerator at 4°C for subsequent sectioning. Paraffin embedding and continuous sectioning of samples were completed on the platform of Wuhan Masp Biotechnology Co. Ltd. A total of 320 paraffin sections that qualified for embryological observation were obtained. The prepared sections were observed under an Olympus BX43 microscope (Olympus, Tokyo, Japan). Complete sections with clear structure were photographed with the Olympus DP80 imaging system, and the photographs were saved.

#### 2.1.2 Determination of Apomixis Rate

Among the numerous florets within a single syconium, only a subset is capable of producing seeds. Thus, the seed setting rate of *F. gasparriniana* was calculated to illustrate its apomictic characteristics. In spring of 2022, 30 syconia at stage B were collected from each plant. The number of normally developed female flowers in each syconium was recorded under an anatomical microscope (Keyence VHX‐5000; Keyence, Tokyo, Japan), and their average number was calculated. In fall, 30 syconia were collected from each plant, and the average number of seeds in each syconium was recorded. Based on the number of female flowers in stage B, the setting rate (Zhang 2021) of female flowers in the apomictic germline was obtained as follows:

$$\begin{array}{cc} \text{Setting rate}\;=\; & \text{Average number of seeds produced in each syconium}/\\ & \text{Average number of stage}\;B\;\text{female flowers in each syconium} \end{array}$$

#### 2.1.3 Seed Anatomy Experiment

Seeds obtained by washing the outer layers of drupes were placed in culture dishes with wet filter papers. For each plant, 100 healthy and plump seeds were germinated. After the endocarp ruptured due to water absorption, seed anatomy was studied under an anatomical microscope. The number of embryos in each seed was recorded. The polyembryony rate was calculated as follows:

Polyembryony rate=Number of seeds withtwoor more embryos/100

### 2.2 Determination of Reproductive Mode of *F. gasparriniana* Populations

#### 2.2.1 Sample Source and Genomic DNA Extraction

All the samples used for simple sequence repeat (SSR) analysis were collected from the natural distribution areas of *F. gasparriniana* in China (Tables S1 and S2). Considering that apomictic plants were mainly found in the Maolan Scenic Area, Libo County, Guizhou Province, the sampling sites were increased near this area and throughout Guizhou (Table S2). The sampling principle was to get as many samples as possible, including at least three mother plants of *F. gasparriniana* from each population (more than 30 m away from each other), three syconia from different branches of each plant, and three plump seeds from each syconium, that is, 1 mother + 9 progeny (1M + 9P) as a sample combination; if insufficient, all were taken. In sample treatment, one or two fresh and healthy leaves were selected from each mother plant, and then the leaf tissue without main veins was immobilized in silica gel for dry preservation. Relevant voucher specimens were deposited in the herbarium of East China Normal University. After the fruit pulp was removed, the seeds were wrapped in absorbent papers for dry preservation. After seeds were germinated and grew into seedlings, genomic DNA was extracted from their leaves by a combination of the cetyltrimethylammonium bromide method and an improved plant DNA extraction method (Li et al. 2013). The fruiting period of samples from four populations in Fengshan County, Guangxi Province, was missed because of the COVID‐19 pandemic, so a 10‐m distance sampling method was adopted in lieu of the sampling of mother and offspring. Thus, 287 samples from 16 populations were genetically identified.

#### 2.2.2 Screening of SSR Loci

Because of high polymorphism and a rapid evolutionary rate, SSR loci are suitable to investigate the genetic differentiation patterns among closely related groups (Zhang 2019). Therefore, SSR loci were used to analyze the genetic relationships between apomictic and sexual reproduction populations. SSR loci had been developed and applied in previous taxonomic studies of *Ficus* (Giraldo et al. 2005; Zhang et al. 2011; Yang and Rannala 2014), and SSR loci suitable for the study of *F. gasparriniana* and its complex populations had been identified (Zhou 2014). These loci were well differentiated among *Ficus* species and showed polymorphism in the same species. Finally, 16 SSR loci with clear amplification bands and high polymorphism were obtained for the genetic identification of *F. gasparriniana*. Loci information is shown in Table S3.

#### 2.2.3 Polymerase Chain Reaction (PCR) Amplification and Detection

Fluorescent PCR combined with capillary electrophoresis separation (short tandem repeat [STR] typing) was used. To reduce experimental time and cost, SSR loci were divided into four groups based on the length range of the amplification primers, and the 5′‐end of each group of SSR primers was labeled by the fluorescent reagent N‐(1‐pyrene)iodoacetamide (PI). The combination method was as follows: pool 1: FP213 + FP328 + FP435 + LMFC13; pool 2: LMFC14 + LMFC15 + LMFC23 + LMFC20; pool 3: LMFC22 + LMFC28 + LMFC35 + LMFC36; and pool 4: LMFC27 + LMFC31 + LMFC32 + LMFC34. After the same sample (SSR loci) was amplified by a set of primers, a mixed sample of the amplified products was sent to Shengong Bioengineering (Shanghai) Co. Ltd., for capillary electrophoresis separation and amplified fragment size analysis. The SSR fluorescent primers were synthesized and provided by Shengong Bioengineering (Shanghai) Co. Ltd. Amplification and analysis of the maternal and progeny samples at the same site were performed in the same batch to reduce experimental errors.

#### 2.2.4 Data Interpretation and Clustering Analysis

Using STR typing, alleles can be distinguished based on the amplified fragment size. If the amplified fragment size at the 16 SSR loci in field‐collected maternal samples was identical with all progeny, and the fluorescence signals in fragment analysis were consistent, then the seeds and progeny of the plant were derived from apomictic reproduction. However, if the amplified fragment size at the 16 loci was not the same between the maternal and progeny samples, or the fluorescence signals in fragment analysis were not consistent, then the reproductive mode was sexual. Because there are no corresponding parent–child samples in the four populations in Fengshan, Guangxi, the SSR genetic relationship among individuals in the whole population was studied. The genetic background of the apomictic reproduction population was generally more consistent.

To further analyze the genetic relationships between apomictic and sexual reproduction populations and between populations from different places, all samples used for SSR genetic identification were also used for SSR‐based genetic cluster analysis. Allelic fragment lengths of all samples were measured, and alleles were marked as 1 and 0, forming a binary dataset. The dataset was analyzed using MEGA5 (Tamura et al. 2011). According to the genetic similarity coefficient, all 287 samples were cluster analyzed by the unweighted pair‐group method with arithmetic means (UPGMA) to construct a clustering tree. The genetic structure was analyzed using STRUCTURE v2.3.4 (50,000 + 150,000, *k* = 1–10, 15 runs/*k*; http://web.stanford.edu/group/pritchardlab/structure.html).

### 2.3 Chromosome Counting and Ploidy Detection of *F. gasparriniana* Populations

#### 2.3.1 Chromosome Preparation

Because of limited source materials, it was not possible to obtain many fresh materials for cytological experiments. Chromosome counting, a direct method to determine plant ploidy by observing chromosome morphology, was used to save time and reduce costs. Five maternal plants (Nos. 40, 44, 45, 46, and 47) and their progeny planted at biological stations were selected for chromosome counting to determine if maternal ploidy was identical to progeny ploidy. *F. gasparriniana* is a woody plant with a small nucleus and high chromosome number; thus, a lot of work would be required to determine plant ploidy. Flow cytometry is fast, simple, and accurate and requires less sample material. Because the fluorescence intensity of the nucleus is proportional to the DNA content after specific fluorescence staining, the ploidy of the test plant can be determined by comparing the DNA content of the plant nucleus with known ploidy samples. Thus, flow cytometry was used to determine the ploidy of the samples from different populations.

The chromosome preparation of *F. gasparriniana* followed the method of Zong and Zhang (2024). The prepared slides were observed under a 100× magnification microscope with oil immersion, and more than 30 cells with a clear division phase were selected. The chromosomes were counted and photographed. Cell chromosome preparation was performed using the root tips of the progeny.

#### 2.3.2 Ploidy Level Determination

Representative maternal and progeny samples were selected from different populations. Among them, the maternal samples were leaves dried by silica gel in the field. A total of 92 progeny samples were fresh young leaves of seedlings germinated from seeds (Table S4). Because of no exact sample of diploid *F. gasparriniana*, the outer reference samples were pre‐detected by chromosome counting and flow cytometry (Wang et al. 2024); these were all 4× “40M” (CK‐40M). PI fluorescence was detected using a MoFL‐XDP high‐speed flow cytometer (Beckman‐Coulter, Brea, CA, USA) with a 70‐μm ceramic nozzle at 60 psi sheath pressure, a laser light source of 488 nm, and a 530‐/40‐nm HQ bandpass filter. At least 500 cells were detected per sample. Using 4× samples as reference, the ploidy was calculated according to the fluorescence intensity as follows:

$$\begin{array}{cc} \text{Ploidy}=\; & \text{Fluorescence mean value of the sample to}\;\mathrm{be}\;\text{measured}\times \\ & \text{Reference sample}/\text{Sample fluorescence mean value} \end{array}$$

Ploidy level was determined at the Key Laboratory of Plant Molecular Physiology, Institute of Botany, Chinese Academy of Sciences, Beijing, China.

## 3 Results and Discussion

### 3.1 Seeds of Unpollinated *F. gasparriniana* Plants Are Derived From Indeterminate Embryos Formed in the Nucellar Tissue

**FIGURE 2:**
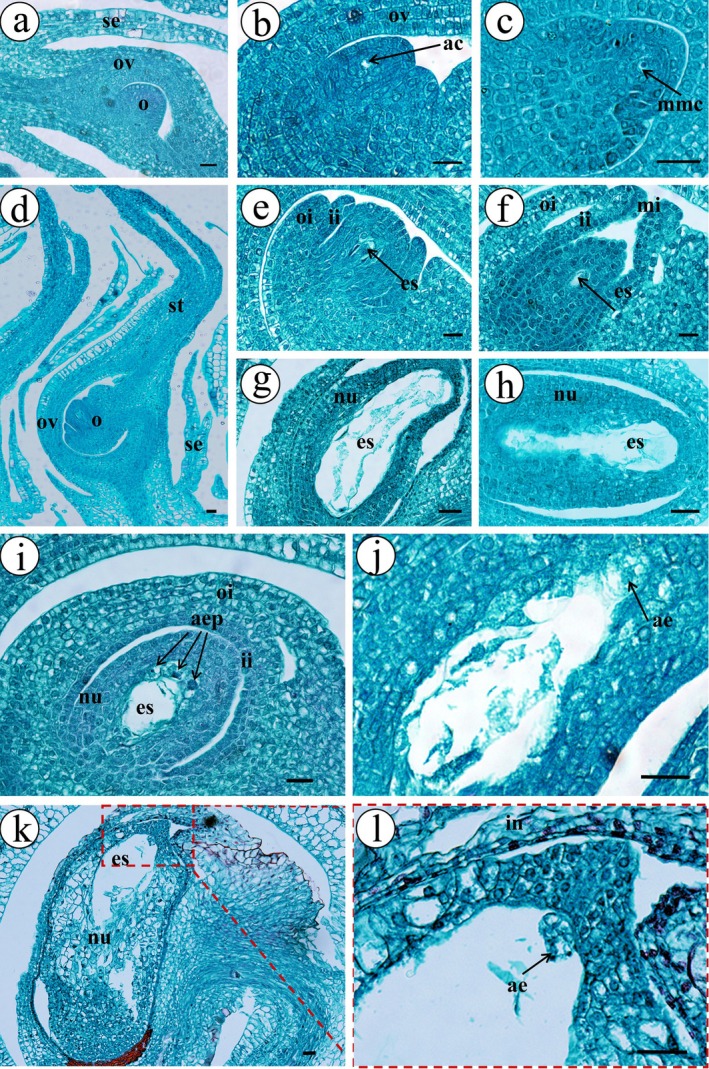
Development of apomictic adventitious embryo in *Ficus gasparriniana*. (a) Ovule primordium; (b) archesporial cell; (c) megaspore mother cells; (d) dividing megaspore mother cell; (e) mononuclear embryo sac; (f) four‐nucleated embryo sac; (g) aborted embryo sac, embryo sac cells can be seen; (h) the aborted embryo sac forms one cavity; (i) nucellar cells forming adventitious embryonic primordia; (j) adventitious embryonic primordium enters the embryo sac; (k) the adventitious embryo absorbs nutrients from the disintegrated nucellus to grow; (l) the developing adventitious embryo. ac, archesporial cell; ae, adventitious embryo; aep, adventitious embryonic primordium; es, embryo sac; ii, inner integument; in, integument; mi, micropyle; mmc, megaspore mother cell; nu, nucellus; o, ovule; oi, outer integument; ov, ovary; se, sepal; st, stigma; scale bars = 20 μm.

Apomixis is of two types: (1) gametophytic apomixis, where the plant embryo is formed within the embryo sac (female gametophyte); and (2) sporophytic apomixis, where embryo development originates from the nucellar or integumentary cells of the sporogenous tissue (proembryo) (Hao and Qiang 2009; Popelka et al. 2019; Mei 2023). In the absence of pollinators, during the development of the ovule primordium to the embryo sac in the stage A ovary of *F. gasparriniana* (Figure S2), the embryo sac undergoes varying degrees of degeneration and disintegration (Figure 2). Even if a mature embryo sac is formed, it gradually disintegrates because of the lack of pollination (Figure 2g,h). When the syconium enters stage B (Figure S2), the nucellar tissue outside a few embryo sacs develops into adventitious embryonic primordia (Figure 2i) and invades the aborted embryo sac (Figure 2j,k). During this process, vacuolation of nucellar tissue outside the embryo sac cavity provides nutrients for nucellar embryo development (Figure 2l). Eventually, the ovule develops into a mature seed with 1–3 indeterminate embryos (Figure S1). This phenomenon differs from apomictic seed production induced by artificial stimulation of the embryo sac (Zdruikovskaya‐Rikhter 1970; Arendt and Kazas 1980), where gametophytic apomictic embryos are influenced by human factors. In contrast, in adventitious embryo reproduction, sporophytic apomictic embryos develop autonomously without any external stimuli.

### 3.2 Apomixis Is Crucial for *F. gasparriniana* Reproduction in Harsh Habitats

The incidence of adventitious embryos (setting rate) of *F. gasparriniana* was about 23% (Table S5). About 38% of the apomictic seeds of *F. gasparriniana* demonstrated polyembryony (Figure S1; Table S6), which increases the number of offspring. Although this study did not obtain data on the sexual reproduction seed setting rate of *F. gasparriniana*, compared with the seed yield of other fig trees under fig wasp pollination, such as 45.45%–63.63% of *F. erecta* var. *beecheyana* (Li et al. 2001), 25.64%–54.13% of *Ficus beipeiensis* (Deng et al. 2014), 7.4%–47.1% of *Ficus virens* var. *sublanceolata* (Wu et al. 2012), and 22.02%–29.1% of *Ficus microcarpa* (Li, Zhang, et al. 1999; Zhang 2021), *F. gasparriniana* reproduces by adventitious embryo reproduction, in which the number of offspring produced by polyembryony can essentially replace the number of offspring produced by sexual reproduction. Hence, apomixis plays an important, and even dominant, role in the natural growth of *F. gasparriniana*.

**FIGURE 3:**
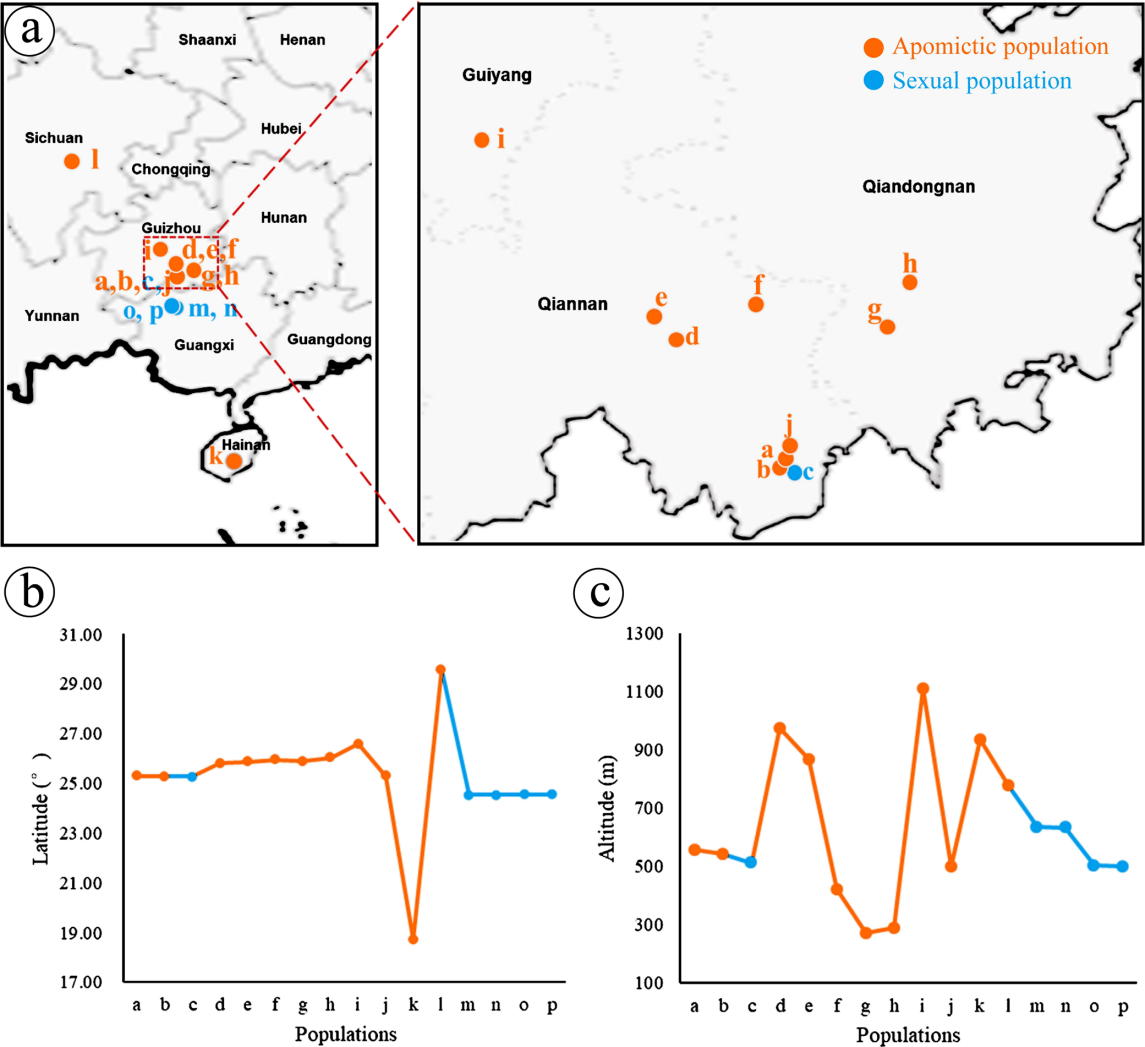
Geographical distribution of *Ficus gasparriniana* populations. (a) Population distribution map; (b) latitude distribution; (c) altitude distribution.

**FIGURE 4:**
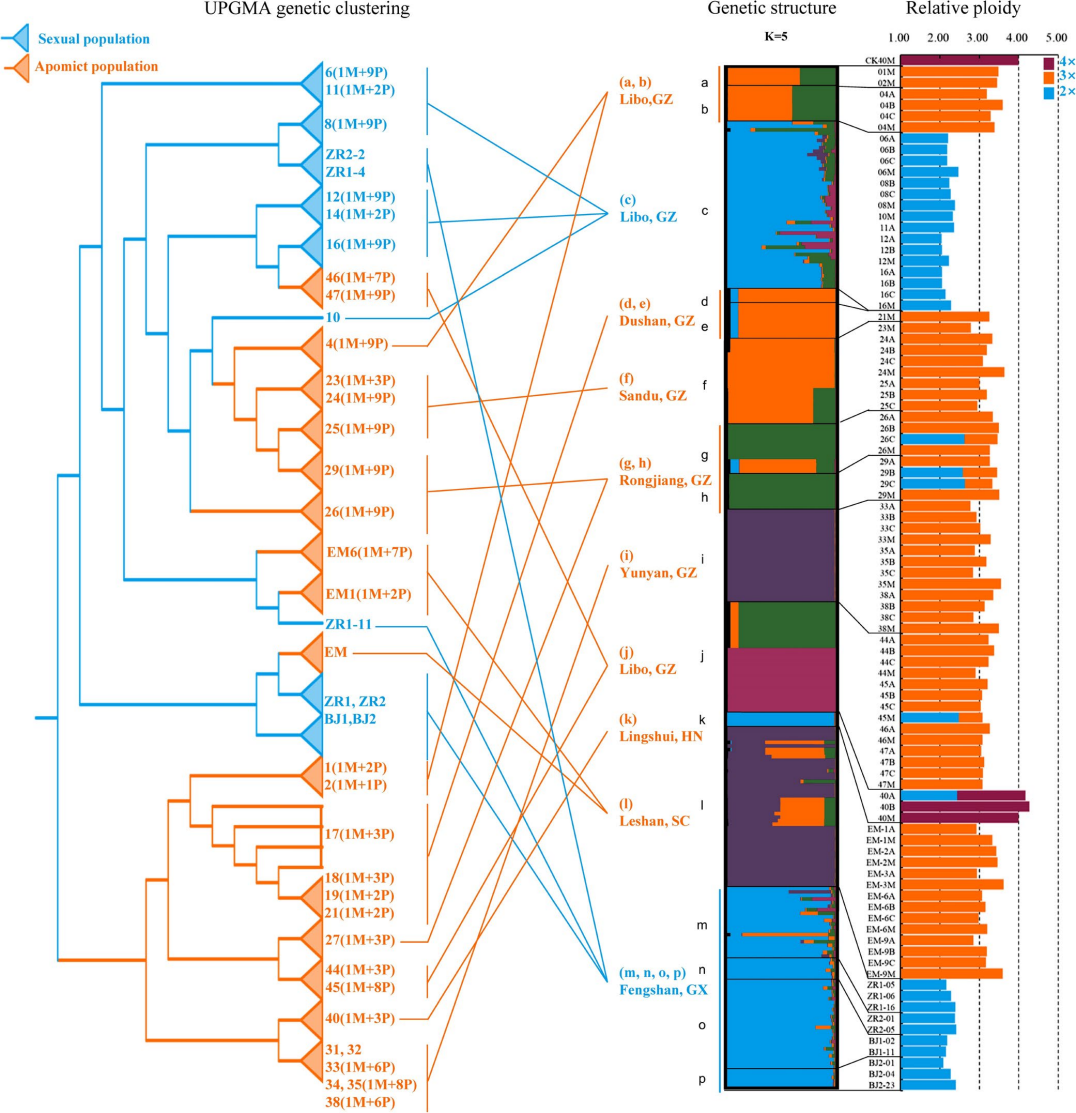
Population genetic structure and relative ploidy of *Ficus gasparriniana*. From left to right: UPGMA genetic clustering, genetic structure, and relative ploidy. M, maternal parent; P, progeny; for example, 4 (1M + 9P) represents one maternal sample and its nine progeny samples of sample group 4.

Molecular markers identified that 11 of 16 natural populations reproduced by apomictic reproduction, while the remaining five populations reproduced sexually by fig wasp pollination (Figures 3 and 4; Tables S7 and S8). Within our sampling range, the apomictic *F. gasparriniana* population was distributed from southern Hainan to central Sichuan (18°43′ N–29°34′ N) (Figure 3a,b), at an altitude of 270–1110 m (Figure 3c), which was more common than the sexual reproduction population. Apomictic lineages were distributed farther north, at higher altitudes, and in a wider range than sexual reproduction lineages (Figure 3). This distribution pattern, also known as geographical parthenogenesis (Vandel 1928), can be found in many plant and animal taxa (Lynch 1984; Bierzychudek 1987).

Apomictic reproduction lineages usually originate from the diploid lineage of sexual reproduction (Thompson and Whitton 2006). In SSR cluster analysis, apomictic *F. gasparriniana* samples clustered on different branches and were closely related to sexual reproduction samples (Figures 3 and 4; Table S7). Apomictic samples from different geographical locations also clustered on different branches, suggesting that apomictic *F. gasparriniana* has multiple origins (Figure 4). At the northern edge and high altitudes, where climatic conditions restrict fig wasp survival, apomictic reproduction is the primary mode for *F. gasparriniana*. The largest population (i) of *F. gasparriniana* is at Qianling Mountain Park in Guiyang (Figures 3a–c and 4; Figure S3a,b), which also has the highest altitude among all sampling sites. No closely related species of *F. gasparriniana* are distributed in Qianling Mountain Park. The consistent genetic background because of apomictic reproduction indicates a stable and long‐term settlement of the population, abandoning the cooperative relationship with fig wasps. The population (l) at Emei Mountain in Sichuan represents the northernmost distribution of *F. gasparriniana* in our sampling range (Figures 3a,b and 4; Figure S3c,d; Table S7). The STRUCTURE results demonstrated that some samples from population (l) have a complex genetic background, which may have originated from hybridization (Figure 4). In the same collection area, female flowers of the closely related species *F. heteromorpha* , which were not pollinated by fig wasps after reaching stage B, droop and their stylar tips turn yellow and wither (Figure S4), leading to the entire syconium falling off without setting fruit, indicating that there are no fig wasps in this region. Evidently, without fig wasp pollinators, *F. gasparriniana* produces offspring by apomictic seeds, increasing the population size and survival rate.

In suitable habitats, *F. gasparriniana* can produce offspring with diverse genetic backgrounds by cooperation with fig wasps. However, in disrupted habitats or where fig wasps are unavailable, apomictic reproduction becomes important to plant survival. *F*. *gasparriniana* reproduced both sexually (with fig wasps) and asexually (apomictic reproduction) in several adjacent populations in Libo County, Guizhou Province (a, b, c, j; Figures 3 and 4; Table S7). Thus, the status of these populations was further investigated during the fig wasp emergence period (April–May) at several collection sites in Libo County, including the Maolan Scenic Area (the original collection site of greenhouse‐introduced individuals), Xiawangtong, and Jiayi. The population (c) in Xiawangtong, located 2.5 km outside the Maolan Scenic Area, was identified by molecular markers and exhibited characteristics of sexual reproduction in all experimental samples (Figure S5). Here, male plants with clear traces in fig wasp emergence were also found (Figure S5d–f). In addition to morphologically diverse *F. gasparriniana* (Figure S5), this site had other fig trees, such as *F. hirta* and *F. formosana* , coexisting in a natural *Pinus massoniana* forest with an undergrowth of dense shrubs, forming a high plant diversity habitat suitable for fig wasp survival and pollination. Therefore, this population reproduces sexually by fig wasp pollination. In contrast, during multiple investigations, no male plants or fig wasps were found in populations (a, b, j), and they were identified as undergoing apomixis in SSR analysis (Figure 4; Table S7). In particular, the *F. gasparriniana* populations (a, j) in the Maolan Scenic Area had a limited distribution range, with only a few individuals growing along the forest edge near pedestrian trails (Figure S3e,f) and affected by human activities. The population (b) in Jiayi, located 3 km away from the Maolan Scenic Area (Figure S3g,h), was sparsely distributed, with only one female plant observed by the roadside. These sparsely distributed or even isolated fig trees might have grown by apomictic reproduction because of occasional long‐distance seed dispersal, the absence of fig wasp pollinators in the local habitat, or environmental stress (Sahu et al. 2013). Therefore, apomictic populations were distributed in higher altitudes, higher latitudes, and larger ranges than sexual reproduction populations, indicating that apomixis is an essential reproductive strategy for natural populations of *F. gasparriniana* to cope with limited fig wasp pollination or habitat stress.

### 3.3 Polyploid of *F. gasparriniana* Is Closely Related to Apomictic Reproduction

The apomictic characteristics of plants are closely related to their ploidy levels. Natural apomictic plant populations are polyploids (Suomalainen et al. 1987; Asker and Jerling 1992), and polyploids are more likely to exhibit apomictic characteristics compared with diploids (Rotreklová and Krahulcová 2016). The ploidy of various population samples was confirmed by flow cytometry combined with chromosome counting and STR typing. All samples exhibiting apomixis were polyploids (3× and 4×), while diploid samples underwent sexual reproduction (Figure 4; Figure S6; Table S9). The genome of polyploids, especially heterozygotes, is unstable and prone to be disordered during cell division, leading to a reduction in somatic chromosomes and mixoploidy in the same individual (Gernand et al. 2005; Sanei et al. 2011; Zhang et al. 2021). In the test samples, such mixoploidy was detected in a small proportion of *F. gasparriniana* (five samples) (Figure 4; Figure S7). Mixoploid plants have been reported in other genera (e.g., *Morus*) of Moraceae (Han et al. 1994; Li et al. 2018).

Furthermore, various ploidy levels exist within the *F. erecta* complex, which includes *F.* *abelii*, *F. periptera*, *F. gasparriniana* (Lu et al. 2017), *F. pandurata* (Wang et al. 2024), *F. erecta* (Shirasawa et al. 2020), *F. iidaiana* (Kusumi et al. 2012), and *F. tannoensis* (Wachi et al. 2016; Wang et al. 2024). Some cultivars of *F. carica* are 3× and 4× (Falistocco 2016). Although we did not investigate whether these species exhibit apomictic characteristics by field surveys or indoor experiments, apomictic characteristics are related to ploidy level (Zhang et al. 2021). Thus, more instances of apomictic reproduction are likely in *Ficus* species, and polyploidy can serve as an important indicator for potential apomictic plants. This study confirmed that flow cytometry and STR typing are effective methods for screening polyploid *F. gasparriniana*, yielding good detection results. While the formation mode of 3× *F. gasparriniana* remains unknown, polyploidy can promote the establishment of apomictic germlines in new ecological niches by altering the overall physiological characteristics and adaptive potential of the plant (Hojsgaard and Hörandl 2019), thereby widely reproducing the population.

### 3.4 Effects of Apomictic Reproduction on Fig–Wasp Coevolution

Because of the specificity of their reproductive organs, fig trees have relied on fig wasps for pollination and reproduction for millions of years (Xu et al. 2011; Cruaud et al. 2012; Zhang et al. 2019). This age‐old pollination relationship, particularly in dioecious fig trees, is demanding and can be disrupted, challenging plant survival. Research on fig–wasp coevolution has primarily focused on sexual reproduction (Herre et al. 1996; Ma et al. 1997; Yang et al. 1997; Jousselin et al. 2003; Rønsted et al. 2005; Yu et al. 2006; Xu and Yang 2008; Compton et al. 2009; Kusumi et al. 2012; Zhang et al. 2020; Wang et al. 2021). We found widespread occurrences of apomictic reproduction in natural *F. gasparriniana* populations. This reproductive mode adds complexity to the fig–wasp coevolution system, and it is worth revisiting how new equilibrium relationships are established in this mutualistic pollination system.

Decreased fig wasp populations and host shifts lead to the transition from sexual reproduction to apomictic reproduction in fig trees. During field investigations, apomictic populations of *F. gasparriniana* often lack male plants, are located in habitats subject to significant human disturbances, and are found at the northern edge of the fig–wasp community, all of which are unsuitable for the survival of fig wasp pollinators (Chen et al. 2018, 2020). Therefore, in sexual reproduction populations in the fig–wasp coevolution system, mutualistic wasp pollinators are susceptible to external influences, leading to population decline or even local extinction (Kjellberg et al. 1987; Jevanandam et al. 2013; Cui 2020). Fig trees that lose their mutualistic pollinators because of the lack of normal pollination are inclined toward apomictic reproduction, which is a compensatory mechanism that plants with long‐term pollination constraints have developed (Wang and Yang 2001). The polyploid characteristics of fig trees further support the development of their apomictic populations (Table S9).

Apomixis is typically a dominant genetic trait, and several different mechanisms have independently evolved in angiosperms (Hörandl and Hojsgaard 2012; León‐Martínez and Vielle‐Calzada 2019). Hybrid origins are common in natural asexual lineages (Hojsgaard and Hörandl 2019), such as *Boechera*, in which almost all natural apomictic species are hybrids (Beck et al. 2012), and the *Sorbus austriaca* complex, in which allopolyploid apomictic species and wasps are highly matched phenologically and morphologically (Hajrudinović‐Bogunić et al. 2023), but mutualistic symbiosis also makes it difficult for fig species to hybridize, compared with other nonspecialist pollinated plants (Jousselin et al. 2003; Moe and Weiblen 2012). However, host switching can break the strict one‐to‐one rule between host and pollinator (Menken 1996; Charleston and Robertson 2002; Renoult et al. 2009), providing opportunities for interspecific hybridization of host fig trees. In fact, interspecific hybridization and host switching are common in *Ficus* (Huang et al. 2019), spanning their long history of coevolution with fig wasps (Wang et al. 2021). In the *F. erecta* complex, *F. erecta* , *F. formosana* , *F. abelii*, *F. pyriformis*, and *F. variolosa* exhibit a range of variations but share the same pollinator, and the complex has unclear species boundaries closely related to numerous host switching events (Su et al. 2022). The origin and development of apomictic reproduction can also be confirmed by interspecific hybridization resulting from host switching of fig wasp pollinators. *F. gasparriniana* trees from Diaoluo Mountain in Hainan (population k) have been found to possess a hybrid background (Wang et al. 2024). Due to the complexity of apomictic reproduction, the potential origin pathway of *F. gasparriniana* remains to be studied. However, the discovery of apomixis in *F. gasparriniana* drives us to reconsider fig–wasp coevolution as well as speciation and evolution in *Ficus*.

Apomictic reproduction drives further evolution of the fig–wasp coevolution system. Fig tree reproduction is influenced by changes in the fig wasp population. Apomictic reproduction in fig trees also affects the development of the fig wasp population. In dioecious fig trees, such as *F. gasparriniana*, after apomictic reproduction partially replaces sexual reproduction, the proportion of male trees in the population will decrease. As only female trees undergo apomictic reproduction, the space provided by fig trees for the survival and reproduction of fig wasps will also decrease. Because fig trees can reproduce without the need for male flowers, the population of fig wasps is likely to decline due to the absence of gall flowers as oviposition sites, which may result in local extinction (Sun et al. 2009; Wang et al. 2010). While asexual reproduction may prevail in harsh habitats on a small scale, a dwindling fig wasp population is not conducive to maintaining sexual reproduction in fig trees. Hence, there will always be a certain proportion of sexual reproductive groups at the species level (Figures 3 and 4; Tables S7 and S8). Through these sexual reproduction populations, the fig–wasp coevolution system will be maintained and driven to establish a new balance. This balance will be gradually shaped by natural selection and coevolutionary processes, reflecting the complexity and dynamics of the long‐term symbiotic relationship between fig trees and their pollinating fig wasps.

In conclusion, we report a case of apomixis in *Ficus* in the absence of pollinating fig wasps. Embryological examination confirmed that this fruiting phenomenon was due to indeterminate embryogenesis or sporophytic apomixis. Polyembryony and polyploidy were common in the sampled populations. Apomixis was common in the wild populations of *F. gasparriniana* and closely linked to sexual reproduction lineages. The sources of apomictic lineages were diverse. Finally, the effects of apomictic reproduction on fig tree survival and the fig–wasp coevolution system were discussed based on the characteristics of apomictic reproduction in *F. gasparriniana*. However, the following issues need to be addressed in the future: (1) whether diploid *F. gasparriniana* has apomictic potential and apomictic polyploids reproduce sexually; (2) how the coevolutionary relationship between *F. gasparriniana* and its wasps can maintain balance in apomictic populations.

## Author Contributions

**Jianhang Zhang:** project administration (equal), resources (equal), software (equal), writing – original draft (equal). **Li Mei:** resources (equal), software (equal), validation (equal), writing – original draft (equal). **Xiaomei Wang:** resources (equal), software (equal), visualization (equal), writing – original draft (equal). **Shuai Liao:** data curation (equal), formal analysis (equal), funding acquisition (equal), software (equal), writing – review and editing (equal). **Hongqing Li:** data curation (equal), funding acquisition (equal), investigation (equal), writing – review and editing (equal). **Zhen Zhang:** conceptualization (equal), data curation (equal), formal analysis (equal), writing – review and editing (equal).

## Conflicts of Interest

The authors declare no conflicts of interest.

## Supporting information


**Appendix S1:** ece372316‐sup‐0001‐AppendixS1.docx.

**Appendix S2:** ece372316‐sup‐0002‐AppendixS2.docx.

## Data Availability

This article has no additional data. Further information and requests about resources and reagents should be directed to the corresponding authors or the first author, Jianhang Zhang (52181300002@stu.ecnu.edu.cn). The materials of apomixis reproduction can also be obtained from Jianhang Zhang.
