# Supplementary material for: Polyembryonic or Apomictic Seeds Enable Fig Trees to Establish New Populations Without Their Pollinating Wasps, a Case Observation in Ficus gasparriniana
Source: Ecol Evol. 2025 Oct 16;15(10):e72316. doi: 10.1002/ece3.72316 (PMC12528958; doi:10.1002/ece3.72316)
Supplement: Supplementary file 1 — Appendix S1: ece372316‐sup‐0001‐AppendixS1.docx. [file ECE3-15-e72316-s001.docx]

Table S1 Detailed collection information of *F. gasparriniana* material involved in this research

| collection number | collector | collecting locations | | | longitude | latitude | altitude (m) | collecting type | analysis |
| --- | --- | --- | --- | --- | --- | --- | --- | --- | --- |
| 20170044* | Zhang Zhen, Zhang Jianhang | Guizhou | Qiannan | Libo | 108°04′19″E | 25°17′49″N | 500 | living plant | SSR、FCM |
| 20170045* | Zhang Zhen, Zhang Jianhang | Guizhou | Qiannan | Libo | 108°04′19″E | 25°17′49″N |  | living plant | SSR、FCM |
| 20170046* | Zhang Zhen, Zhang Jianhang | Guizhou | Qiannan | Libo | 108°04′19″E | 25°17′49″N |  | living plant | SSR、FCM |
| 20170047* | Zhang Zhen, Zhang Jianhang | Guizhou | Qiannan | Libo | 108°04′19″E | 25°17′49″N |  | living plant | SSR、FCM |
| 20190040* | Wang Xiaomei, Zhang Jianhang | Hainan |  | Lingshui | 109°52′01.97″E | 18°43′41.00″N | 932 | living plant | SSR、FCM |
| 20220001 | Li Hongqing, Mei Li, Li Jiecheng | Guizhou | Qiannan | Libo | 108°4'25.76″E | 25°17'35.45″N | 557 | leaves and seeds | SSR、FCM |
| 20220002 | Li Hongqing, Mei Li, Li Jiecheng | Guizhou | Qiannan | Libo | 108°4'25.76″E | 25°17'35.45″N | 557 | leaves and seeds | SSR、FCM |
| 20220004 | Li Hongqing, Mei Li, Li Jiecheng | Guizhou | Qiannan | Libo | 108°4'17.59″E | 25°16'45.07″N | 542 | leaves and seeds | SSR、FCM |
| 20220006 | Li Hongqing, Mei Li, Li Jiecheng | Guizhou | Qiannan | Libo | 108°5'14.16″E | 25°16'6.68″N | 512 | leaves and seeds | SSR、FCM |
| 20220008 | Li Hongqing, Mei Li, Li Jiecheng | Guizhou | Qiannan | Libo | 108°5'13.94″E | 25°16'5.64″N | 514 | leaves and seeds | SSR、FCM |
| 20220010 | Li Hongqing, Mei Li, Li Jiecheng | Guizhou | Qiannan | Libo | 108°5'15.43″E | 25°16'8.44″N | 506 | leaves | SSR、FCM |
| 20220011 | Li Hongqing, Mei Li, Li Jiecheng | Guizhou | Qiannan | Libo | 108°5'16.32″E | 25°16'8″N | 505 | leaves and seeds | SSR、FCM |
| 20220012 | Li Hongqing, Mei Li, Li Jiecheng | Guizhou | Qiannan | Libo | 108°5'15.43″E | 25°16'8.44″N | 506 | leaves and seeds | SSR、FCM |
| 20220014 | Li Hongqing, Mei Li, Li Jiecheng | Guizhou | Qiannan | Libo | 108°5'18.36″E | 25°16'9.7″N | 503 | leaves and seeds | SSR |
| 20220016 | Li Hongqing, Mei Li, Li Jiecheng | Guizhou | Qiannan | Libo | 108°5'21.83″E | 25°16'8.33″N | 505 | leaves and seeds | SSR、FCM |
| 20220017 | Li Hongqing, Mei Li, Li Jiecheng | Guizhou | Qiannan | Dushan | 107°32'42.42″E | 25°47'58.3″N | 976 | leaves and seeds | SSR |
| 20220018 | Li Hongqing, Mei Li, Li Jiecheng | Guizhou | Qiannan | Dushan | 107°29'47.42″E | 25°52'24.28″N | 867 | leaves and seeds | SSR |
| 20220019 | Li Hongqing, Mei Li, Li Jiecheng | Guizhou | Qiannan | Dushan | 107°29'51.09″E | 25°52'24.33″N | 865 | leaves and seeds | SSR |
| 20220021 | Li Hongqing, Mei Li, Li Jiecheng | Guizhou | Qiannan | Dushan | 107°30'10.47″E | 25°52'11.35″N | 894 | leaves and seeds | SSR、FCM |
| 20220023 | Li Hongqing, Mei Li, Li Jiecheng | Guizhou | Qiannan | Sandu | 107°49'56.69″E | 25°56'45.62″N | 422 | leaves and seeds | SSR、FCM |
| 20220024 | Li Hongqing, Mei Li, Li Jiecheng | Guizhou | Qiannan | Sandu | 107°49'54″E | 25°56'42.59″N | 421 | leaves and seeds | SSR、FCM |
| 20220025 | Li Hongqing, Mei Li, Li Jiecheng | Guizhou | Qiannan | Sandu | 107°49'54″E | 25°56'42.59″N | 421 | leaves and seeds | SSR、FCM |
| 20220026 | Li Hongqing, Mei Li, Li Jiecheng | Guizhou | Qiandongnan | Rongjiang | 108°27'50.14″E | 25°52'43.67″N | 271 | leaves and seeds | SSR、FCM |
| 20220027 | Li Hongqing, Mei Li, Li Jiecheng | Guizhou | Qiandongnan | Rongjiang | 108°27'47.4″E | 25°52'39.64″N | 281 | leaves and seeds | SSR |
| 20220029 | Li Hongqing, Mei Li, Li Jiecheng | Guizhou | Qiandongnan | Rongjiang | 108°33'12.22″E | 26°2'21.62″N | 288 | leaves and seeds | SSR、FCM |
| 20220031 | Li Hongqing, Mei Li, Li Jiecheng | Guizhou | Guiyang | Yunyan | 106°41'39.52″E | 26°35'25.1″N | 1111 | leaves | SSR |
| 20220032 | Li Hongqing, Mei Li, Li Jiecheng | Guizhou | Guiyang | Yunyan | 106°41'40.52″E | 26°35'34.73″N | 1108 | leaves | SSR |
| 20220033 | Mei Li | Guizhou | Guiyang | Yunyan | 106°41'42.34″E | 26°35'25.2″N | 1116 | leaves and seeds | SSR、FCM |
| 20220034 | Mei Li | Guizhou | Guiyang | Yunyan | 106°41'41.96″E | 26°35'33.95″N | 1112 | leaves | SSR |
| 20220035 | Mei Li | Guizhou | Guiyang | Yunyan | 106°41'41.48″E | 26°35'34.71″N | 1116 | leaves and seeds | SSR、FCM |
| 20220038 | Mei Li | Guizhou | Guiyang | Yunyan | 106°41'39.95″E | 26°35'35.16″N | 1123 | leaves and seeds | SSR、FCM |
| EM1 | Li Hongqing | Sichuan | Leshan | Emei | 103°24′18.33″E | 29°34′5.08″N | 780 | leaves and seeds | SSR、FCM |
| EM2 | Li Hongqing | Sichuan | Leshan | Emei | 103°23′54.07″E | 29°34′11.84″N | 777 | leaves and seeds | SSR、FCM |
| EM3 | Li Hongqing | Sichuan | Leshan | Emei | 103°23′46.98″E | 29°34′13.21″N | 738 | leaves and seeds | SSR、FCM |
| EM4 | Li Hongqing | Sichuan | Leshan | Emei | 103°23′46.99″E | 29°34′13.22″N | 738 | leaves and seeds | SSR |
| EM6 | Li Hongqing | Sichuan | Leshan | Emei | 103°23′39.98″E | 29°34′18.77″N | 706 | leaves and seeds | SSR、FCM |
| EM7 | Li Hongqing | Sichuan | Leshan | Emei | 103°23′35.79″E | 29°34′28.78″N | 786 | leaves and seeds | SSR |
| EM8 | Li Hongqing | Sichuan | Leshan | Emei | 103°23′35.80″E | 29°34′28.79″N | 786 | leaves and seeds | SSR |
| EM9 | Li Hongqing | Sichuan | Leshan | Emei | 103°23′5.91″E | 29°35′5.24″N | 897 | leaves and seeds | SSR |
| EM10 | Li Hongqing | Sichuan | Leshan | Emei | 103°23′3.67″E | 29°35′8.64″N | 862 | leaves and seeds | SSR、FCM |
| EM11 | Li Hongqing | Sichuan | Leshan | Emei | 103°23′1.96″E | 29°35′11.59″N | 857 | leaves | SSR |
| ZR1-01 | Li Hongqing, Mei Li, Zhang Chenlu | Guangxi | Hechi | Fengshan | 107°13′28″E | 24°32′2″N | 635 | leaves | SSR |
| ZR1-02 | Li Hongqing, Mei Li, Zhang Chenlu | Guangxi | Hechi | Fengshan | 107°13′28″E | 24°32′2″N |  | leaves | SSR |
| ZR1-03 | Li Hongqing, Mei Li, Zhang Chenlu | Guangxi | Hechi | Fengshan | 107°13′28″E | 24°32′2″N |  | leaves | SSR |
| ZR1-04 | Li Hongqing, Mei Li, Zhang Chenlu | Guangxi | Hechi | Fengshan | 107°13′28″E | 24°32′2″N |  | leaves | SSR |
| ZR1-05 | Li Hongqing, Mei Li, Zhang Chenlu | Guangxi | Hechi | Fengshan | 107°13′28″E | 24°32′2″N |  | leaves | SSR、FCM |
| ZR1-06 | Li Hongqing, Mei Li, Zhang Chenlu | Guangxi | Hechi | Fengshan | 107°13′28″E | 24°32′2″N |  | leaves | SSR、FCM |
| ZR1-07 | Li Hongqing, Mei Li, Zhang Chenlu | Guangxi | Hechi | Fengshan | 107°13′28″E | 24°32′2″N |  | leaves | SSR |
| ZR1-08 | Li Hongqing, Mei Li, Zhang Chenlu | Guangxi | Hechi | Fengshan | 107°13′28″E | 24°32′2″N |  | leaves | SSR |
| ZR1-09 | Li Hongqing, Mei Li, Zhang Chenlu | Guangxi | Hechi | Fengshan | 107°13′28″E | 24°32′2″N |  | leaves | SSR |
| ZR1-10 | Li Hongqing, Mei Li, Zhang Chenlu | Guangxi | Hechi | Fengshan | 107°13′28″E | 24°32′2″N |  | leaves | SSR |
| ZR1-11 | Li Hongqing, Mei Li, Zhang Chenlu | Guangxi | Hechi | Fengshan | 107°13′28″E | 24°32′2″N |  | leaves | SSR |
| ZR1-12 | Li Hongqing, Mei Li, Zhang Chenlu | Guangxi | Hechi | Fengshan | 107°13′28″E | 24°32′2″N |  | leaves | SSR |
| ZR1-13 | Li Hongqing, Mei Li, Zhang Chenlu | Guangxi | Hechi | Fengshan | 107°13′28″E | 24°32′2″N |  | leaves | SSR |
| ZR1-14 | Li Hongqing, Mei Li, Zhang Chenlu | Guangxi | Hechi | Fengshan | 107°13′28″E | 24°32′2″N |  | leaves | SSR |
| ZR1-15 | Li Hongqing, Mei Li, Zhang Chenlu | Guangxi | Hechi | Fengshan | 107°13′28″E | 24°32′2″N |  | leaves | SSR |
| ZR1-16 | Li Hongqing, Mei Li, Zhang Chenlu | Guangxi | Hechi | Fengshan | 107°13′28″E | 24°32′2″N |  | leaves | SSR、FCM |
| ZR1-17 | Li Hongqing, Mei Li, Zhang Chenlu | Guangxi | Hechi | Fengshan | 107°13′28″E | 24°32′2″N |  | leaves | SSR |
| ZR1-18 | Li Hongqing, Mei Li, Zhang Chenlu | Guangxi | Hechi | Fengshan | 107°13′28″E | 24°32′2″N |  | leaves | SSR |
| ZR1-19 | Li Hongqing, Mei Li, Zhang Chenlu | Guangxi | Hechi | Fengshan | 107°13′28″E | 24°32′2″N |  | leaves | SSR |
| ZR1-20 | Li Hongqing, Mei Li, Zhang Chenlu | Guangxi | Hechi | Fengshan | 107°13′28″E | 24°32′2″N |  | leaves | SSR |
| ZR2-01 | Li Hongqing, Mei Li, Zhang Chenlu | Guangxi | Hechi | Fengshan | 107°13′27″E | 24°32′9″N | 633 | leaves | SSR、FCM |
| ZR2-02 | Li Hongqing, Mei Li, Zhang Chenlu | Guangxi | Hechi | Fengshan | 107°13′27″E | 24°32′9″N |  | leaves | SSR |
| ZR2-03 | Li Hongqing, Mei Li, Zhang Chenlu | Guangxi | Hechi | Fengshan | 107°13′27″E | 24°32′9″N |  | leaves | SSR |
| ZR2-04 | Li Hongqing, Mei Li, Zhang Chenlu | Guangxi | Hechi | Fengshan | 107°13′27″E | 24°32′9″N |  | leaves | SSR |
| ZR2-05 | Li Hongqing, Mei Li, Zhang Chenlu | Guangxi | Hechi | Fengshan | 107°13′27″E | 24°32′9″N |  | leaves | SSR、FCM |
| ZR2-06 | Li Hongqing, Mei Li, Zhang Chenlu | Guangxi | Hechi | Fengshan | 107°13′27″E | 24°32′9″N |  | leaves | SSR |
| BJ1-01 | Li Hongqing, Mei Li, Zhang Chenlu | Guangxi | Hechi | Fengshan | 107°13′18″E | 24°32′57″N | 503 | leaves | SSR |
| BJ1-02 | Li Hongqing, Mei Li, Zhang Chenlu | Guangxi | Hechi | Fengshan | 107°13′18″E | 24°32′57″N |  | leaves | SSR、FCM |
| BJ1-03 | Li Hongqing, Mei Li, Zhang Chenlu | Guangxi | Hechi | Fengshan | 107°13′18″E | 24°32′57″N |  | leaves | SSR |
| BJ1-04 | Li Hongqing, Mei Li, Zhang Chenlu | Guangxi | Hechi | Fengshan | 107°13′18″E | 24°32′57″N |  | leaves | SSR |
| BJ1-05 | Li Hongqing, Mei Li, Zhang Chenlu | Guangxi | Hechi | Fengshan | 107°13′18″E | 24°32′57″N |  | leaves | SSR |
| BJ1-06 | Li Hongqing, Mei Li, Zhang Chenlu | Guangxi | Hechi | Fengshan | 107°13′18″E | 24°32′57″N |  | leaves | SSR |
| BJ1-07 | Li Hongqing, Mei Li, Zhang Chenlu | Guangxi | Hechi | Fengshan | 107°13′18″E | 24°32′57″N |  | leaves | SSR |
| BJ1-08 | Li Hongqing, Mei Li, Zhang Chenlu | Guangxi | Hechi | Fengshan | 107°13′18″E | 24°32′57″N |  | leaves | SSR |
| BJ1-09 | Li Hongqing, Mei Li, Zhang Chenlu | Guangxi | Hechi | Fengshan | 107°13′18″E | 24°32′57″N |  | leaves | SSR |
| BJ1-10 | Li Hongqing, Mei Li, Zhang Chenlu | Guangxi | Hechi | Fengshan | 107°13′18″E | 24°32′57″N |  | leaves | SSR |
| BJ1-11 | Li Hongqing, Mei Li, Zhang Chenlu | Guangxi | Hechi | Fengshan | 107°13′18″E | 24°32′57″N |  | leaves | SSR、FCM |
| BJ1-12 | Li Hongqing, Mei Li, Zhang Chenlu | Guangxi | Hechi | Fengshan | 107°13′18″E | 24°32′57″N |  | leaves | SSR |
| BJ1-13 | Li Hongqing, Mei Li, Zhang Chenlu | Guangxi | Hechi | Fengshan | 107°13′18″E | 24°32′57″N |  | leaves | SSR |
| BJ1-14 | Li Hongqing, Mei Li, Zhang Chenlu | Guangxi | Hechi | Fengshan | 107°13′18″E | 24°32′57″N |  | leaves | SSR |
| BJ1-15 | Li Hongqing, Mei Li, Zhang Chenlu | Guangxi | Hechi | Fengshan | 107°13′18″E | 24°32′57″N |  | leaves | SSR |
| BJ1-16 | Li Hongqing, Mei Li, Zhang Chenlu | Guangxi | Hechi | Fengshan | 107°13′18″E | 24°32′57″N |  | leaves | SSR |
| BJ1-17 | Li Hongqing, Mei Li, Zhang Chenlu | Guangxi | Hechi | Fengshan | 107°13′18″E | 24°32′57″N |  | leaves | SSR |
| BJ1-18 | Li Hongqing, Mei Li, Zhang Chenlu | Guangxi | Hechi | Fengshan | 107°13′18″E | 24°32′57″N |  | leaves | SSR |
| BJ1-19 | Li Hongqing, Mei Li, Zhang Chenlu | Guangxi | Hechi | Fengshan | 107°13′18″E | 24°32′57″N |  | leaves | SSR |
| BJ1-20 | Li Hongqing, Mei Li, Zhang Chenlu | Guangxi | Hechi | Fengshan | 107°13′18″E | 24°32′57″N |  | leaves | SSR |
| BJ1-21 | Li Hongqing, Mei Li, Zhang Chenlu | Guangxi | Hechi | Fengshan | 107°13′18″E | 24°32′57″N |  | leaves | SSR |
| BJ1-22 | Li Hongqing, Mei Li, Zhang Chenlu | Guangxi | Hechi | Fengshan | 107°13′18″E | 24°32′57″N |  | leaves | SSR |
| BJ1-23 | Li Hongqing, Mei Li, Zhang Chenlu | Guangxi | Hechi | Fengshan | 107°13′18″E | 24°32′57″N |  | leaves | SSR、FCM |
| BJ1-24 | Li Hongqing, Mei Li, Zhang Chenlu | Guangxi | Hechi | Fengshan | 107°13′18″E | 24°32′57″N |  | leaves | SSR |
| BJ1-25 | Li Hongqing, Mei Li, Zhang Chenlu | Guangxi | Hechi | Fengshan | 107°13′18″E | 24°32′57″N |  | leaves | SSR |
| BJ1-26 | Li Hongqing, Mei Li, Zhang Chenlu | Guangxi | Hechi | Fengshan | 107°13′18″E | 24°32′57″N |  | leaves | SSR |
| BJ2-01 | Li Hongqing, Mei Li, Zhang Chenlu | Guangxi | Hechi | Fengshan | 107°13′17″E | 24°32′58″N | 500 | leaves | SSR、FCM |
| BJ2-02 | Li Hongqing, Mei Li, Zhang Chenlu | Guangxi | Hechi | Fengshan | 107°13′17″E | 24°32′58″N |  | leaves | SSR |
| BJ2-03 | Li Hongqing, Mei Li, Zhang Chenlu | Guangxi | Hechi | Fengshan | 107°13′17″E | 24°32′58″N |  | leaves | SSR |
| BJ2-04 | Li Hongqing, Mei Li, Zhang Chenlu | Guangxi | Hechi | Fengshan | 107°13′17″E | 24°32′58″N |  | leaves | SSR、FCM |
| BJ2-05 | Li Hongqing, Mei Li, Zhang Chenlu | Guangxi | Hechi | Fengshan | 107°13′17″E | 24°32′58″N |  | leaves | SSR |

Note: * denotes plants still cultivated in the greenhouse of Biological Experiment Station of East China Normal University.

Table S2 Information for the population and samples

| **population ID** | **collecting locations** | | | | **sample groups** | **sampling number** | **longitude and latitude** | **altitude (m)** |
| --- | --- | --- | --- | --- | --- | --- | --- | --- |
| **a** | Guizhou | Libo | Maolan | Jinshidong | 1(1M+2P)  2(1M+1P) | 5 | 108°4'25.76″E  25°17'35.45″N | 557 |
| **b** | Guizhou | Libo | Maolan | Jiayi | 4(1M+9P) | 10 | 108°4'17.59″E  25°16'45.07″N | 542 |
| **c** | Guizhou | Libo | Maolan | xiawangtong | 6(1M+9P)  8(1M+9P),10  11(1M+2P)  12(1M+9P)  14(1M+2P)  16(1M+9P) | 47 | 108°5'21.83″E  25°16'8.33″N | 512 |
| **d** | Guizhou | Dushan | Baiquan | Xiaohecun | 17(1M+3P) | 4 | 107°32'42.42″E  25°47'58.3″N | 976 |
| **e** | Guizhou | Dushan | Mawan | Piaolicun | 18(1M+3P)  19(1M+2P)  21(1M+2P) | 10 | 107°29'51.09″E  25°52'24.33″N | 867 |
| **f** | Guizhou | Sandu | Bakai | Jiashaishuiku | 23(1M+3P)  24(1M+9P)  25(1M+9P) | 24 | 107°49'56.69″E  25°56'45.62″N | 422 |
| **g** | Guizhou | Rongjiang | Bakai | Dujiangcun | 26(1M+9P)  27(1M+3P) | 14 | 108°27'50.14″E  25°52'43.67″N | 271 |
| **h** | Guizhou | Rongjiang | Zhongcheng | Lexiangcun | 29(1M+9P) | 10 | 108°33'12.22″E  26°2'21.62″N | 288 |
| **i** | Guizhou | Guiyang | Yunyan | Qianling mountain | 31, 32  33(1M+6P)  34,35(1M+8P)  38(1M+6P) | 26 | 106°41'39.52″E  26°35'25.1″N | 1110 |
| **j** | Guizhou | Libo | Maolan | Loudousenlin | 44(1M+3P)  45(1M+8P)  46(1M+7P)  47(1M+9P) | 31 | 108°04′19.24″E  25°17′49.27″N | 500 |
| **k** | Hainan | Lingshui | Diaoluoshan | Shenshu | 40(1M+3P) | 4 | 109°52′03.95″E  18°43′37.99″N | 937 |
| **l** | Sichuan | Leshan | Emei | Emei mountain | EM1(1M+3P)  EM2(1M+3P)  EM3(1M+3P)  EM4(1M+3P)  EM6(1M+7P)  EM7(1M+8P)  EM8(1M+6P)  EM10(1M+4P)  EM11 | 46 | 103°24′18.33″E  29°34′5.08″N | 780 |
| **m** | Guangxi | Fengshan | Changzhou | Zhouruocun 1 | ZR1(20) | 20 | 107°13′28.04″E  24°32′2.4″N | 635 |
| **n** | Guangxi | Fengshan | Changzhou | Zhouruocun 2 | ZR2(6) | 6 | 107°13′27.45″E  24°32′9.4″N | 633 |
| **o** | Guangxi | Fengshan | Changzhou | Banjuncun 1 | BJ1(25) | 25 | 107°13′18″E  24°32′57″N | 503 |
| **p** | Guangxi | Fengshan | Changzhou | Banjuncun 2 | BJ2(5) | 5 | 107°13′17″E  24°32′58″N | 500 |

Note: The numbers outside brackets in the sample group column are the sample group number (corresponding to the last two digits of the collection number in Table S1), the numbers in brackets represent the sample number, **M,** maternal parent. **P**, progeny, such as 4(1M+9P) represents the 1 maternal sample and its 9 progeny samples of sample group 4.

Table S3 Primers sequence and related information of 16 SSR loci

| loci | microsatellite definition | primer sequence | fluorescent primers | length range | references |
| --- | --- | --- | --- | --- | --- |
| FP213 | (CT)_7_…(CT)_9_ | F: CATGCAACAAACTTCCTTAC | 5’<TAMRA> | 187-209 | Zhang et al., 2011 |
|  |  | R: GTACTAGGATTGTTCGTGCT |  |  |  |
| FP328 | (CT)_11_…(CT)_14_ | F: TCAATTCTTTTCGCTATCTC | 5’<6-FAM> | 131-167 | Zhang et al., 2011 |
|  |  | R: GAAGCTACGTTGTGCTTTAT |  |  |  |
| FP435 | (AG)_10_…(AG)_16_ | F: GTTATTACAAGGTTTGGTCG | 5’<ROX> | 157-179 | Zhang et al., 2011 |
|  |  | R: GCAACAAACTTCCTTACATT |  |  |  |
| LMFC13 | (GA)_28_ | F: CCTCTTTCTCTCTCTTAATTTT | 5’<HEX> | 253-305 | Giraldo et al., 2005 |
|  |  | R: TTTATCAAACCCACTGATTC |  |  |  |
| LMFC14 | (GA)_16_ | F: CAAAACTCACACCAATAATC | 5’<TAMRA> | 194-228 | Giraldo et al., 2005 |
|  |  | R: TAATCTGCAAAAAGATGACTA |  |  |  |
| LMFC15 | (TC)_22_ | F: CGGAGAAAGATTTAGAATTTG | 5’<6-FAM> | 203-217 | Giraldo et al., 2005 |
|  |  | R: ATTCCAGAGACGAAAGGTCT |  |  |  |
| LMFC20 | (AAG)_9_(AG)_18_ | F: ATGGAGGCTTAGATAGAAAT | 5’<HEX> | 122-155 | Giraldo et al., 2005 |
|  |  | R: ACAACACAAAAAGAAATATCA |  |  |  |
| LMFC22 | (AG)_13_ | F: ATCACGATATAGGTGTTTTAAT | 5’<TAMRA> | 270-298 | Giraldo et al., 2005 |
|  |  | R: AGACTTGTAATTTTGATTCCT |  |  |  |
| LMFC23 | (AG)_20_ | F: TTTCGTGTCTAACGATCAAAAA | 5’<ROX> | 120-130 | Giraldo et al., 2005 |
|  |  | R: CTCCCATCTCCAACTCCATC |  |  |  |
| LMFC27 | (TG)_17_(AG)_6_ | F: ATTTCTTCAACTTTTGTAATGA | 5’<TAMRA> | 186-224 | Giraldo et al., 2005 |
|  |  | R: CCTTTTGTCTACATATACCTTT |  |  |  |
| LMFC28 | (CT)_14_ | F: TGATTCCTTTTACTTGTAGATT | 5’<6-FAM> | 175-215 | Giraldo et al., 2005 |
|  |  | R: AAGACATTGAGACATACCAG |  |  |  |
| LMFC31 | (GA)_15_ | F: GTAAAATGAAAATTGGAGTATT | 5’<6-FAM> | 219-255 | Giraldo et al., 2005 |
|  |  | R: TTGAAGATATTGTTGTATGCT |  |  |  |
| LMFC32 | (GA)_23_ | F: GAAAGAAAGTCGAATAATGTA | 5’<ROX> | 165-221 | Giraldo et al., 2005 |
|  |  | R: TATAAAGAGGGTGGTCTTAGT |  |  |  |
| LMFC34 | (GA)_17_ | F: GTTACAAAGTACAGGTAAGCA | 5’<HEX> | 232-258 | Giraldo et al., 2005 |
|  |  | R: GTATTGGATCTTGATTATGTTT |  |  |  |
| LMFC35 | (CT)_11_ | F: CTCAACCCCACCATTTTAAC | 5’<ROX> | 244-286 | Giraldo et al., 2005 |
|  |  | R: AGCTCTTTGTTGCTTCGATT |  |  |  |
| LMFC36 | (CT)_18_ | F: GACTCCTACACCATCAAAGG | 5’<HEX> | 220-266 | Giraldo et al., 2005 |
|  |  | R: CTTCACGTTGTTCCTGTTGT |  |  |  |

Table S4 Flow cytometry raw data

| ID | first | | | | second | | | | third | | | | fourth | | | |
| --- | --- | --- | --- | --- | --- | --- | --- | --- | --- | --- | --- | --- | --- | --- | --- | --- |
|  | count | mean | CV | relative ploidy | count | mean | CV | relative ploidy | count | mean | CV | relative ploidy | counts | mean | CV | relative ploidy |
| CK-40M | 1200 | 80.24 | 3.86 | 4.00 | 1543 | 51.25 | 4.48 | 4.00 | 969 | 63.39 | 3.66 | 4.00 | 416 | 72.14 | 2.63 | 4.00 |
| 24A | 1586 | 66.79 | 5.25 | 3.33 |  |  |  |  | 1604 | 44.84 | 5.29 | 2.83 |  |  |  |  |
| 24B | 1078 | 63.98 | 3.01 | 3.19 |  |  |  |  | 1284 | 45.05 | 4.97 | 2.84 |  |  |  |  |
| 24C | 1446 | 61.91 | 4.72 | 3.09 |  |  |  |  | 1474 | 51.16 | 4.94 | 3.23 |  |  |  |  |
| 35A | 1123 | 57.93 | 6.18 | 2.89 |  |  |  |  | 968 | 41.77 | 4.26 | 2.64 |  |  |  |  |
| 35B | 1168 | 63.73 | 5.55 | 3.18 |  |  |  |  | 930 | 46.13 | 5.15 | 2.91 |  |  |  |  |
| 35C | 1264 | 57.00 | 5.95 | 2.84 |  |  |  |  | 756 | 45.44 | 4.74 | 2.87 |  |  |  |  |
| 40M | 1200 | 80.24 | 3.86 | 4.00 |  |  |  |  | 969 | 63.39 | 3.66 | 4.00 | 416 | 72.14 | 2.63 | 4.00 |
| 40A | 1105 | 48.98 | 5.01 | 2.44 |  |  |  |  | 860 | 63.26 | 3.98 | 3.99 |  |  |  |  |
|  | 1865 | 83.62 | 4.38 | 4.17 |  |  |  |  | 665 | 38.51 | 3.51 | 2.43 |  |  |  |  |
| 40B | 1380 | 85.70 | 3.82 | 4.27 |  |  |  |  | 660 | 63.96 | 3.29 | 4.04 |  |  |  |  |
| 44M | 1668 | 58.31 | 5.26 | 2.91 |  |  |  |  | 426 | 47.54 | 3.46 | 3.00 |  |  |  |  |
| 44A | 1676 | 64.75 | 5.47 | 3.23 |  |  |  |  | 1343 | 53.26 | 4.40 | 3.36 |  |  |  |  |
| 44B | 2852 | 67.80 | 4.26 | 3.38 |  |  |  |  | 884 | 51.40 | 2.91 | 3.24 |  |  |  |  |
| 44C | 1843 | 64.95 | 4.63 | 3.24 |  |  |  |  | 1089 | 52.50 | 4.49 | 3.31 |  |  |  |  |
| 45M | 1249 | 49.75 | 4.78 | 2.48 |  |  |  |  | 240 | 42.21 | 3.38 | 2.66 |  |  |  |  |
|  | 765 | 61.89 | 5.23 | 3.09 |  |  |  |  | 532 | 49.04 | 3.61 | 3.09 |  |  |  |  |
| 45A | 1214 | 64.39 | 4.59 | 3.21 |  |  |  |  |  |  |  |  |  |  |  |  |
| 45B | 1403 | 61.61 | 4.75 | 3.07 |  |  |  |  | 746 | 45.88 | 3.14 | 2.90 |  |  |  |  |
| 45C | 1573 | 60.96 | 5.19 | 3.04 |  |  |  |  |  |  |  |  | 615 | 49.46 | 2.64 | 2.74 |
| 46M | 717 | 61.81 | 5.01 | 3.08 |  |  |  |  |  |  |  |  | 480 | 58.12 | 3.43 | 3.22 |
| 46A | 2675 | 65.50 | 5.71 | 3.27 |  |  |  |  |  |  |  |  |  |  |  |  |
| 47M | 3678 | 61.79 | 5.43 | 3.08 |  |  |  |  |  |  |  |  | 968 | 50.59 | 3.13 | 2.81 |
| 47A | 2156 | 61.23 | 5.33 | 3.05 |  |  |  |  |  |  |  |  | 833 | 52.99 | 2.75 | 2.94 |
| 47B | 3220 | 62.64 | 6.15 | 3.12 |  |  |  |  |  |  |  |  | 2003 | 55.78 | 3.69 | 3.09 |
| 47C | 1457 | 61.99 | 6.09 | 3.09 |  |  |  |  |  |  |  |  | 667 | 50.79 | 3.07 | 2.82 |
| 4A |  |  |  |  | 673 | 40.93 | 4.61 | 3.19 |  |  |  |  |  |  |  |  |
| 4B |  |  |  |  | 1183 | 44.72 | 4.12 | 3.49 |  |  |  |  |  |  |  |  |
| 4C |  |  |  |  | 571 | 42.15 | 3.74 | 3.29 |  |  |  |  |  |  |  |  |
| 6A |  |  |  |  | 1505 | 28.29 | 5.50 | 2.21 |  |  |  |  |  |  |  |  |
| 6B |  |  |  |  | 1580 | 28.06 | 5.10 | 2.19 |  |  |  |  |  |  |  |  |
| 6C |  |  |  |  | 1101 | 27.96 | 3.57 | 2.18 |  |  |  |  |  |  |  |  |
| 8B |  |  |  |  |  |  |  |  | 1214 | 32.85 | 5.29 | 2.24 |  |  |  |  |
| 8C |  |  |  |  |  |  |  |  | 1463 | 33.30 | 4.91 | 2.27 |  |  |  |  |
| 11A |  |  |  |  |  |  |  |  | 1280 | 34.60 | 4.87 | 2.36 |  |  |  |  |
| 12A |  |  |  |  |  |  |  |  | 1330 | 29.98 | 5.36 | 2.05 |  |  |  |  |
| 12B |  |  |  |  |  |  |  |  | 728 | 30.10 | 4.53 | 2.05 |  |  |  |  |
| 16A |  |  |  |  |  |  |  |  | 1557 | 30.16 | 5.46 | 2.06 |  |  |  |  |
| 16B |  |  |  |  |  |  |  |  | 1105 | 30.06 | 5.20 | 2.05 |  |  |  |  |
| 16C |  |  |  |  |  |  |  |  | 1274 | 31.44 | 4.84 | 2.15 |  |  |  |  |
| 25A |  |  |  |  |  |  |  |  | 1118 | 43.96 | 4.22 | 3.00 |  |  |  |  |
| 25B |  |  |  |  |  |  |  |  | 1187 | 46.66 | 4.19 | 3.18 |  |  |  |  |
| 25C |  |  |  |  |  |  |  |  | 924 | 43.14 | 4.46 | 2.94 |  |  |  |  |
| 26A |  |  |  |  |  |  |  |  | 948 | 49.00 | 3.98 | 3.34 |  |  |  |  |
| 26B |  |  |  |  |  |  |  |  | 1448 | 51.23 | 4.04 | 3.50 |  |  |  |  |
| 26C |  |  |  |  |  |  |  |  | 497 | 38.42 | 3.33 | 2.62 |  |  |  |  |
|  |  |  |  |  |  |  |  |  | 1674 | 50.70 | 4.13 | 3.46 |  |  |  |  |
| 29A |  |  |  |  |  |  |  |  | 1184 | 51.82 | 3.68 | 3.27 |  |  |  |  |
| 29B |  |  |  |  |  |  |  |  | 286 | 37.75 | 3.87 | 2.58 |  |  |  |  |
|  |  |  |  |  |  |  |  |  | 353 | 50.53 | 3.27 | 3.45 |  |  |  |  |
| 29C |  |  |  |  |  |  |  |  | 242 | 38.52 | 3.67 | 2.63 |  |  |  |  |
|  |  |  |  |  |  |  |  |  | 763 | 48.73 | 3.69 | 3.33 |  |  |  |  |
| 33A |  |  |  |  |  |  |  |  | 612 | 37.78 | 3.98 | 2.58 |  |  |  |  |
| 33B |  |  |  |  |  |  |  |  | 1063 | 42.93 | 3.57 | 2.93 |  |  |  |  |
| 33C |  |  |  |  |  |  |  |  | 586 | 44.30 | 3.33 | 3.02 |  |  |  |  |
| 38A |  |  |  |  |  |  |  |  | 526 | 49.20 | 2.99 | 3.36 |  |  |  |  |
| 38B |  |  |  |  |  |  |  |  | 759 | 45.98 | 2.87 | 3.14 |  |  |  |  |
| 38C |  |  |  |  |  |  |  |  | 578 | 41.76 | 3.22 | 2.85 |  |  |  |  |
| EM1A |  |  |  |  |  |  |  |  | 1040 | 42.86 | 4.54 | 2.93 |  |  |  |  |
| EM2A |  |  |  |  |  |  |  |  | 1136 | 50.31 | 4.28 | 3.43 |  |  |  |  |
| EM3A |  |  |  |  |  |  |  |  | 1063 | 43.02 | 5.22 | 2.94 |  |  |  |  |
| EM6A |  |  |  |  |  |  |  |  | 1403 | 44.91 | 4.52 | 3.07 |  |  |  |  |
| EM6B |  |  |  |  |  |  |  |  | 1285 | 46.19 | 3.89 | 3.15 |  |  |  |  |
| EM6C |  |  |  |  |  |  |  |  | 461 | 43.62 | 3.27 | 2.98 |  |  |  |  |
| EM9A |  |  |  |  |  |  |  |  | 622 | 41.85 | 4.52 | 2.86 |  |  |  |  |
| EM9B |  |  |  |  |  |  |  |  | 864 | 46.66 | 3.52 | 3.18 |  |  |  |  |
| EM9C |  |  |  |  |  |  |  |  | 975 | 46.42 | 3.34 | 3.17 |  |  |  |  |
| 1M |  |  |  |  |  |  |  |  |  |  |  |  | 655 | 62.85 | 3.90 | 3.48 |
| 2M |  |  |  |  |  |  |  |  |  |  |  |  | 1286 | 62.27 | 5.02 | 3.45 |
| 4M |  |  |  |  |  |  |  |  |  |  |  |  | 1086 | 61.13 | 3.83 | 3.39 |
| 6M |  |  |  |  |  |  |  |  |  |  |  |  | 813 | 44.61 | 5.53 | 2.47 |
| 8M |  |  |  |  |  |  |  |  |  |  |  |  | 881 | 43.00 | 4.71 | 2.38 |
| 10M |  |  |  |  |  |  |  |  |  |  |  |  | 1185 | 42.10 | 5.25 | 2.33 |
| 12M |  |  |  |  |  |  |  |  |  |  |  |  | 336 | 40.33 | 4.53 | 2.24 |
| 16M |  |  |  |  |  |  |  |  |  |  |  |  | 968 | 41.14 | 4.98 | 2.28 |
| 21M |  |  |  |  |  |  |  |  |  |  |  |  | 951 | 58.71 | 3.44 | 3.26 |
| 23M |  |  |  |  |  |  |  |  |  |  |  |  | 1275 | 44.87 | 5.13 | 2.49 |
| 24M |  |  |  |  |  |  |  |  |  |  |  |  | 979 | 65.52 | 3.20 | 3.63 |
| 26M |  |  |  |  |  |  |  |  |  |  |  |  | 766 | 58.95 | 4.39 | 3.27 |
| 29M |  |  |  |  |  |  |  |  |  |  |  |  | 1236 | 63.13 | 3.98 | 3.50 |
| 33M |  |  |  |  |  |  |  |  |  |  |  |  | 780 | 59.39 | 3.43 | 3.29 |
| 35M |  |  |  |  |  |  |  |  |  |  |  |  | 371 | 64.07 | 4.13 | 3.55 |
| 38M |  |  |  |  |  |  |  |  |  |  |  |  | 474 | 63.05 | 3.70 | 3.50 |
| EM1M |  |  |  |  |  |  |  |  |  |  |  |  | 1026 | 60.15 | 3.90 | 3.34 |
| EM2M |  |  |  |  |  |  |  |  |  |  |  |  | 206 | 62.49 | 3.89 | 3.46 |
| EM3M |  |  |  |  |  |  |  |  |  |  |  |  | 187 | 65.13 | 4.82 | 3.61 |
| EM6M |  |  |  |  |  |  |  |  |  |  |  |  | 691 | 57.79 | 3.50 | 3.20 |
| EM9M |  |  |  |  |  |  |  |  |  |  |  |  | 1407 | 64.77 | 4.66 | 3.59 |
| BJ1-02 |  |  |  |  |  |  |  |  |  |  |  |  | 978 | 39.46 | 5.97 | 2.19 |
| BJ1-11 |  |  |  |  |  |  |  |  |  |  |  |  | 1087 | 38.83 | 5.10 | 2.15 |
| BJ2-23 |  |  |  |  |  |  |  |  |  |  |  |  | 667 | 43.46 | 5.06 | 2.41 |
| BJ2-01 |  |  |  |  |  |  |  |  |  |  |  |  | 498 | 37.67 | 4.63 | 2.09 |
| BJ2-04 |  |  |  |  |  |  |  |  |  |  |  |  | 626 | 41.06 | 4.39 | 2.28 |
| ZR1-05 |  |  |  |  |  |  |  |  |  |  |  |  | 1808 | 39.14 | 4.93 | 2.17 |
| ZR1-06 |  |  |  |  |  |  |  |  |  |  |  |  | 908 | 41.12 | 5.46 | 2.28 |
| ZR1-16 |  |  |  |  |  |  |  |  |  |  |  |  | 852 | 43.10 | 4.62 | 2.39 |
| ZR2-01 |  |  |  |  | 1980 | 33.98 | 6.92 | 2.39 |  |  |  |  |  |  |  |  |
| ZR2-05 |  |  |  |  |  |  |  |  |  |  |  |  | 954 | 43.64 | 4.79 | 2.42 |

Note: The sample number corresponds to the last two digits of the collection number. **Count**, the number of cells used for flow cytometry analysis. **Mean**, means of fluorescence. **CV**, the percentage of coefficient of variation, which is used to evaluate the effect of nuclear extraction. The normal range for this group is CV value below 8 containing phenols and other impurities.

Table S5 The seed setting rate of female flowers in apomictic syconia

| **samples** | **mean number of female flowers in** a **syconium (n=30)** | **mean number of seeds in a syconium (n=30)** | **setting rate** |
| --- | --- | --- | --- |
| 40M | 56.87±8.06 | 11.48±2.91 | 20.19% |
| 45M | 81.63±4.78 | 19.59±4.85 | 24.00% |
| 46M | 89.37±7.13 | 23.30±5.85 | 26.07% |
| average | - | - | 23.42% |

Table S6 Polyembryonic rate statistics

| **samples** | **seed number** | **single embryo number** | **single embryo rate** | **double embryo number** | **double embryo rate** | **three embryo number** | **three embryo rate** | **Total number of polyembryos** | **polyembryos rate** |
| --- | --- | --- | --- | --- | --- | --- | --- | --- | --- |
| 40M | 100 | 65 | 65.00% | 28 | 28.00% | 7 | 7.00% | 35 | 35.00% |
| 44M | 100 | 58 | 58.00% | 35 | 35.00% | 7 | 7.00% | 42 | 42.00% |
| 45M | 100 | 62 | 62.00% | 30 | 30.00% | 8 | 8.00% | 38 | 38.00% |
| total | 300 | 185 | 61.67% | 93 | 31.00% | 22 | 7.33% | 174 | 38.33% |

| **population ID** | **sample groups** | **consistent parent-child genetic information (Yes/No)** | **Apomictic/Sexual** |
| --- | --- | --- | --- |
| **a** | 1(1M+2P), 2(1M+1P) | Yes | Apomictic |
| **b** | 4(1M+9P) | Yes | Apomictic |
| **c** | 6(1M+9P), 8(1M+9P)  10, 11(1M+2P), 12(1M+9P), 14(1M+2P), 16(1M+9P) | No | Sexual |
| **d** | 17(1M+3P) | Yes | Apomictic |
| **e** | 18(1M+3P), 19(1M+2P), 21(1M+2P) | Yes | Apomictic |
| **f** | 23(1M+3P), 24(1M+9P),  25(1M+9P) | Yes | Apomictic |
| **g** | 26(1M+9P), 27(1M+3P) | Yes | Apomictic |
| **h** | 29(1M+9P) | Yes | Apomictic |
| **i** | 31, 32, 33(1M+6P)  34, 35(1M+8P), 38(1M+6P) | Yes | Apomictic |
| **j** | 44(1M+3P), 45(1M+8P)  46(1M+7P), 47(1M+9P) | Yes | Apomictic |
| **k** | 40(1M+3P) | Yes | Apomictic |
| **l** | EM1(1M+3P), EM2(1M+3P)  EM3(1M+3P), EM4(1M+3P)  EM6(1M+7P), EM7(1M+8P)  EM8(1M+6P), EM10(1M+4P)  EM11 | Yes | Apomictic |
| **m** | ZR1(20) | - | Sexual |
| **n** | ZR1(6) | - | Sexual |
| **o** | BJ1(25) | - | Sexual |
| **p** | BJ2(5) | - | Sexual |

Table S7 Apomixis identification of various groups of parent-child samples

Note: The numbers outside brackets in the sample group column are the sample group number (corresponding to the last two digits of the collection number in Table S1), the numbers in brackets represent the sample number, **M**, maternal parent. **P**, progeny, such as 4(1M+9P) represents the 1 maternal sample and its 9 progeny samples of sample group 4.

Table S8 Genotypes of 16 SSR loci in each sample group

| **P-ID** | **SG**  **/Primer** | **SSR locus genotype** | | | | | | | | | | | | | | | |
| --- | --- | --- | --- | --- | --- | --- | --- | --- | --- | --- | --- | --- | --- | --- | --- | --- | --- |
|  |  | **FP213** | **FP328** | **FP435** | **L13** | **L14** | **L15** | **L23** | **L20** | **L22** | **L28** | **L35** | **L36** | **L27** | **L31** | **L32** | **L34** |
| a | 1M | 199/203 | 151/155 | 169/173 | 279/287 | 213 | 206/212 | 130 | 136/150 | 284 | 177/189 | 274/280/284 | 248 | 196 | 227 | -9 | 248 |
|  | 1A | 199/203 | 151/155 | 169/173 | 279/287 | 213 | 206/212 | 130 | 136/150 | 284 | 177/189 | 274/280/284 | 248 | 196 | 227 | -9 | 248 |
|  | 1B | 199/203 | 151/155 | 169/173 | 279/287 | 213 | 206/212 | 130 | 136/150 | 284 | 177/189 | 274/280/284 | 248 | 196 | 227 | -9 | 248 |
|  | 2M | 199/203 | 151/155 | 169/173 | 279/287 | 213 | 206/212 | 130 | 136/150 | 284 | 177/189 | 274/280/284 | 248 | 196 | 227 | -9 | 248 |
|  | 2A | 199/203 | 151/155 | 169/173 | 279/287 | 213 | 206/212 | 130 | 136/150 | 284 | 177/189 | 274/280/284 | 248 | 196 | 227 | -9 | 248 |
| b | 4M | 199/203 | 151/155 | 169/173 | 279/287 | 213 | 206/212 | 130 | 136/150 | 284 | 177/189 | 258/262 | 246/248 | 196 | 227 | 187/191 | 248 |
|  | 4A1 | 199/203 | 151/155 | 169/173 | 279/287 | 213 | 206/212 | 130 | 136/150 | 284 | 177/189 | 258/262 | 246/248 | 196 | 227 | 187/191 | 248 |
|  | 4A2 | 199/203 | 151/155 | 169/173 | 279/287 | 213 | 206/212 | 130 | 136/150 | 284 | 177/189 | 258/262 | 246/248 | 196 | 227 | 187/191 | 248 |
|  | 4A3 | 199/203 | 151/155 | 169/173 | 279/287 | 213 | 206/212 | 130 | 136/150 | 284 | 177/189 | 258/262 | 246/248 | 196 | 227 | 187/191 | 248 |
|  | 4B1 | 199/203 | 151/155 | 169/173 | 279/287 | 213 | 206/212 | 130 | 136/150 | 284 | 177/189 | 258/262 | 246/248 | 196 | 227 | 187/191 | 248 |
|  | 4B2 | 199/203 | 151/155 | 169/173 | 279/287 | 213 | 206/212 | 130 | 136/150 | 284 | 177/189 | 258/262 | 246/248 | 196 | 227 | 187/191 | 248 |
|  | 4B3 | 199/203 | 151/155 | 169/173 | 279/287 | 213 | 206/212 | 130 | 136/150 | 284 | 177/189 | 258/262 | 246/248 | 196 | 227 | 187/191 | 248 |
|  | 4C1 | 199/203 | 151/155 | 169/173 | 279/287 | 213 | 206/212 | 130 | 136/150 | 284 | 177/189 | 258/262 | 246/248 | 196 | 227 | 187/191 | 248 |
|  | 4C2 | 199/203 | 151/155 | 169/173 | 279/287 | 213 | 206/212 | 130 | 136/150 | 284 | 177/189 | 258/262 | 246/248 | 196 | 227 | 187/191 | 248 |
|  | 4C3 | 199/203 | 151/155 | 169/173 | 279/287 | 213 | 206/212 | 130 | 136/150 | 284 | 177/189 | 258/262 | 246/248 | 196 | 227 | 187/191 | 248 |
| c | 6M | 199/205 | 151/157 | 169/175 | -9 | 209/213 | 206/212 | 130 | -9 | 284 | -9 | 258/260 | 246/250 | 196 | 227 | 191/203 | 248 |
|  | 6A1 | 199 | 151 | 169/175 | -9 | 213 | 206/212 | 130/138 | -9 | 284 | 197/201 | 258/260 | 246 | 196 | 227 | 191 | 248 |
|  | 6A2 | 199/205 | 151 | 169/175 | -9 | 213 | 206 | 130/138 | -9 | 284 | -9 | 258 | 246 | 196 | 227 | 191 | 248 |
|  | 6A3 | 205 | 151 | 175 | -9 | 213 | 206/212 | 130/138 | -9 | 284 | -9 | 260 | 250 | 196 | 227 | 191 | 248 |
|  | 6B1 | 199 | 151/157 | 169 | -9 | 213/215 | 212 | 130 | -9 | 284 | -9 | 260 | 246/250 | 196 | 227 | 187/203 | 248 |
|  | 6B2 | 205 | 151 | 175 | -9 | 209/213 | 206 | 130/138 | -9 | 280/284 | -9 | 258 | 246 | 196 | 227 | 187/203 | 248 |
|  | 6B3 | 199/205 | 151/157 | 175 | -9 | 209/213 | 206 | 130/138 | -9 | 284 | 197/201 | 258 | 246 | 196 | 227 | 187/191 | 248 |
|  | 6C1 | 205 | 151 | 175 | -9 | 209/215 | 206/212 | 130 | -9 | 280/284 | -9 | 260/266 | 246 | 196 | 227 | 191/203 | 248 |
|  | 6C2 | 199/205 | 151/157 | 175 | -9 | 209/213 | 206 | 130/138 | -9 | 280/284 | -9 | 258 | 250 | 196 | 227 | 191 | 248 |
|  | 6C3 | 199 | 151/157 | 169 | -9 | 209/213 | 206 | 130/138 | -9 | 280/284 | -9 | 258 | 246 | 196 | 227 | 191 | 248 |
|  | 8M | 203 | 157 | 173 | 281 | 213 | 206 | 130 | 136/150 | 284 | -9 | 258/266 | -9 | 196 | 227 | 191 | 248 |
|  | 8A1 | 203 | 151/157 | 173 | 281 | 209/213 | 206 | 130 | 136/150 | 280/284 | -9 | 258 | 246 | 196 | 227 | 191 | 248 |
|  | 8A2 | 203 | 151/157 | 173 | 281 | 209/213 | 206 | 130 | 136/150 | 280/284 | -9 | 258 | -9 | 196 | 227 | 191 | 248 |
|  | 8A3 | 199/203 | 151/157 | 169/173 | 281/287 | 213/215 | 206 | 130 | 150 | 284 | -9 | 258 | -9 | 196 | 227 | 191 | 248 |
|  | 8B1 | 199/203 | 157 | 169/173 | 281 | 213 | 206 | 130 | 136/150 | 284 | -9 | 258/266 | -9 | 196 | 227 | 191 | 248 |
|  | 8B2 | 203 | 157 | 173 | 281 | 213 | 206 | 130 | 136/150 | 284 | -9 | 258/266 | -9 | 196 | 227 | 191 | 248 |
|  | 8B3 | 203 | 151/157 | 173 | 281 | 213 | 206 | 130 | 150 | 284 | -9 | 258/266 | -9 | 196 | 227 | 191 | 248 |
|  | 8C1 | 199/203 | 157 | 169/173 | 281 | 213 | 206 | 130 | 150 | 284 | -9 | 258 | -9 | 196/208 | 227 | 191 | 248 |
|  | 8C2 | 203 | 151/157 | 173 | 281 | 213 | 206 | 130 | 136/150 | 284 | -9 | 258/266 | -9 | 196/208 | 227 | 191 | 248 |
|  | 8C3 | 203 | 157 | 173 | 281 | 213 | 206 | 130 | 136/150 | 284 | -9 | 258/266 | -9 | 196/208 | 227 | 191 | 248 |
|  | 10(male) | 199/203 | 151/155 | 169/173 | 287 | 209/215 | 206 | 130 | 136/150 | 280/284 | 177/183 | 258 | 248/250 | 196 | 227 | 209/231 | 248 |
|  | 10(re-male) | 199/203 | 151/155 | 169/173 | 287 | 209/215 | 206 | 130 | 136/150 | 280/284 | 177/183 | 258 | 248/250 | 196 | 227 | 209/231 | 248 |
|  | 11M | 199 | 151/157 | 169 | 279 | 209 | 206/212 | 130 | -9 | 280/284 | -9 | 272/278 | 246/250 | 196 | 227 | 191 | 248 |
|  | 11A | 199/205 | 151 | 169/175 | 279 | 209 | 206/212 | 130 | -9 | 280 | -9 | 272 | 246/250 | 196 | 227 | 191 | 248 |
|  | 11B | 199/203 | 151/157 | 169/173 | 279 | 209/213 | 206 | 130 | -9 | 280/284 | -9 | 278 | 246 | 196 | 227 | 191 | 248 |
|  | 12M | 203 | 153/157 | 173 | 281 | 209/213 | 206 | 130 | 150 | 280/284 | 177/189 | 258/266 | 242/246 | 196 | 227 | 187/205 | 248 |
|  | 12A1 | 199/203 | 157 | 169/173 | 281 | 209/213 | 206 | 130 | 144/150 | 280/284 | 189 | 258 | 242/248 | 196 | 227 | 205/213 | 248 |
|  | 12A2 | 199/203 | 151/153 | 169/173 | 281 | 213 | 206 | 130 | 144/150 | 280/284 | 177/189 | 258/266 | 242/248 | 196 | 227 | 187/205 | 248 |
|  | 12A3 | 203 | 151/157 | 173 | 281 | 209/213 | 206 | 130 | 144/150 | 280/284 | 177/189 | 266 | 242/248 | 196 | 227 | 187/191 | 248 |
|  | 12B1 | 199/203 | 151/157 | 169/173 | 281/283 | 213/215 | 206 | 130 | 150 | 284 | 177/189 | 258 | 246 | 196 | 227 | 205 | 248 |
|  | 12B2 | 203 | 153/157 | 173 | 281/283 | 209/213 | 206 | 130 | 150 | 280/284 | 177/189 | 258 | 246 | 196 | 227 | 205 | 248 |
|  | 12B3 | 199/203 | 153/157 | 173 | 281/283 | 213/215 | 206 | 130 | 136/150 | 284 | 183/189 | 258 | 242/248 | 196 | 227 | 187 | 248 |
|  | 12C1 | 203 | 151/157 | 173 | 281 | 213/215 | 206 | 130 | 136/150 | 284 | 189 | 258/266 | 242/248 | 196 | 227 | 191/205 | 248 |
|  | 12C2 | 199/203 | 157 | 169/173 | 281 | 213 | 206 | 130 | 150 | 284 | 177/183 | 258 | 246/248 | 196 | 227 | 205 | 248 |
|  | 12C3 | 199/203 | 151/157 | 169/173 | 281 | 209/213 | 206 | 130 | 150 | 280/284 | 177/183 | 258 | 246 | 196/202 | 227 | 187 | 248 |
|  | 14M | 203/205 | 151 | 173 | 281/285 | 209/213 | 206 | 130 | 140 | 284 | 189 | 258/266 | 246 | 196/202 | 227 | 191 | 248 |
|  | 14A1 | 203 | 157 | 173 | 281/285 | 209 | 206 | 130 | 140 | 280/284 | 183/189 | 266 | 246 | 196 | 227 | 191 | 248 |
|  | 14A2 | 203/205 | 151/157 | 173 | 281 | 209 | 206 | 130 | 140 | 280/284 | 183/189 | 258/266 | 246 | 196/202 | 227 | 187/191 | 248 |
|  | 16M | 205 | 151/157 | 175 | 281 | 209 | 206 | 130 | 136/150 | 284 | 183/189 | 258 | 250 | 196 | 225/227 | 191 | 248 |
|  | 16A1 | 205 | 153/157 | 175 | 277/285 | 209 | 206 | 130 | 136 | 284 | 183/189 | 254/258 | 246/250 | 196 | 227 | 191 | 248 |
|  | 16A2 | 205 | 153/157 | 175 | 281/285 | 209/213 | 206 | 130 | 136 | 280/284 | 189 | 254/258 | 246/250 | 196 | 225/227 | 191 | 248 |
|  | 16A3 | 203/205 | 151/157 | 175 | 277/281 | 209/213 | 206 | 130 | 136 | 280/284 | 189 | 258 | 250 | 196 | 225/227 | 191 | 248 |
|  | 16B1 | 199/205 | 157 | 169/175 | 281 | 209/213 | 206 | 130 | 136/150 | 284 | 177/189 | 258 | 246/250 | 196 | 227 | 191 | 244/248 |
|  | 16B2 | 203/205 | 151 | 175 | 281/283 | 209/213 | 206 | 130 | 136/150 | 284 | 183/189 | 258/266 | 248/250 | 196 | 227 | 187/191 | 248 |
|  | 16B3 | 203/205 | 157 | 175 | 281/283 | 209 | 206 | 130 | 136/150 | 280/284 | 183/189 | 258 | 250 | 196 | 227 | 187/191 | 248 |
|  | 16C1 | 199/205 | 151/157 | 169/175 | 281/283 | 209/213 | 206 | 130 | 136/150 | 284 | 183/189 | 258/266 | 248/250 | 196 | 227 | 187/191 | 244/248 |
|  | 16C2 | 203/205 | 151/157 | 175 | 281 | 209/213 | 206 | 130 | 150 | 284 | 177/189 | 258 | 250 | 196 | 225/227 | 191 | 244/248 |
|  | 16C3 | 203/205 | 157 | 175 | 281 | 209 | 206 | 130/138 | 136/150 | 280/284 | 177/189 | 258 | 250 | 196 | 227 | 191 | 244/248 |
| d | 17M | 199/203 | 151/155 | 169/173 | 279/281/285 | 213 | 208/212 | 130/138 | 136 | 284 | 189 | 258/274/284 | 230/248/250 | 196 | 227 | -9 | 248 |
|  | 17A | 199/203 | 151/155 | 169/173 | 279/281/285 | 213 | 208/212 | 130/138 | 136 | 284 | 189 | 258/274/284 | 230/248/250 | 196 | 227 | -9 | 248 |
|  | 17B | 199/203 | 151/155 | 169/173 | 279/281/285 | 213 | 208/212 | 130/138 | 136 | 284 | 189 | 258/274/284 | 230/248/250 | 196 | 227 | -9 | 248 |
|  | 17C | 199/203 | 151/155 | 169/173 | 279/281/285 | 213 | 208/212 | 130/138 | 136 | 284 | 189 | 258/274/284 | 230/248/250 | 196 | 227 | -9 | 248 |
| e | 18M | 199/203 | 151/155 | 169/173 | 279/281/285 | 213 | 208/212 | 130/138 | 136 | 284 | 189 | 258/274/284 | 230/248/250 | 196 | 227 | -9 | 248 |
|  | 18A | 199/203 | 151/155 | 169/173 | 279/281/285 | 213 | 208/212 | 130/138 | 136 | 284 | 189 | 258/274/284 | 230/248/250 | 196 | 227 | -9 | 248 |
|  | 18B | 199/203 | 151/155 | 169/173 | 279/281/285 | 213 | 208/212 | 130/138 | 136 | 284 | 189 | 258/274/284 | 230/248/250 | 196 | 227 | -9 | 248 |
|  | 18C | 199/203 | 151/155 | 169/173 | 279/281/285 | 213 | 208/212 | 130/138 | 136 | 284 | 189 | 258/274/284 | 230/248/250 | 196 | 227 | -9 | 248 |
|  | 19M | 199/203 | 151/155 | 169/173 | 279/281/285 | 213 | 208/212 | 130/138 | 136 | 284 | 189 | 258/274/284 | 230/248/250 | 196 | 227 | -9 | 248 |
|  | 19A | 199/203 | 151/155 | 169/173 | 279/281/285 | 213 | 208/212 | 130/138 | 136 | 284 | 189 | 258/274/284 | 230/248/250 | 196 | 227 | -9 | 248 |
|  | 19B | 199/203 | 151/155 | 169/173 | 279/281/285 | 213 | 208/212 | 130/138 | 136 | 284 | 189 | 258/274/284 | 230/248/250 | 196 | 227 | -9 | 248 |
|  | 21M | 199/203 | 151/155 | 169/173 | 279/281/285 | 213 | 208/212 | 130/138 | 136 | 284 | 189 | 258/274/284 | 230/248/250 | 196 | 227 | -9 | 248 |
|  | 21A | 199/203 | 151/155 | 169/173 | 279/281/285 | 213 | 208/212 | 130/138 | 136 | 284 | 189 | 258/274/284 | 230/248/250 | 196 | 227 | -9 | 248 |
|  | 21BC | 199/203 | 151/155 | 169/173 | 279/281/285 | 213 | 208/212 | 130/138 | 136 | 284 | 189 | 258/274/284 | 230/248/250 | 196 | 227 | -9 | 248 |
| f | 23M | 199/203 | 153/155 | 169/173 | 281/287 | 205/209/213 | 206/212 | 130 | 136/140/150 | 276/280/284 | 183/189 | 258/260/270 | 242/248 | 196 | 227 | 191 | 248 |
|  | 23A | 199/203 | 153/155 | 169/173 | 281/287 | 205/209/213 | 206/212 | 130 | 136/140/150 | 276/280/284 | 183/189 | 258/260/270 | 242/248 | 196 | 227 | 191 | 248 |
|  | 23B | 199/203 | 153/155 | 169/173 | 281/287 | 205/209/213 | 206/212 | 130 | 136/140/150 | 276/280/284 | 183/189 | 258/260/270 | 242/248 | 196 | 227 | 191 | 248 |
|  | 23C | 199/203 | 153/155 | 169/173 | 281/287 | 205/209/213 | 206/212 | 130 | 136/140/150 | 276/280/284 | 183/189 | 258/260/270 | 242/248 | 196 | 227 | 191 | 248 |
|  | 24M | 199/203 | 151/155 | 169/173 | 279/287 | 205/209/213 | 206/212 | 130 | 136/140/150 | 276/280/284 | 183/189 | 258/260/270 | 242/248 | 196 | 227 | 191/233 | 248 |
|  | 24M | 199/203 | 151/155 | 169/173 | 279/287 | 205/209/213 | 206/212 | 130 | 136/140/150 | 276/280/284 | 183/189 | 258/260/270 | 242/248 | 196 | 227 | 191/233 | 248 |
|  | 24A1 | 199/203 | 151/155 | 169/173 | -9 | 205/209/213 | 206/212 | 130 | 136/140/150 | 276/280/284 | 183/189 | 258/260/270 | 242/248 | 196 | 227 | 191/233 | 248 |
|  | 24A1 | 199/203 | 151/155 | 169/173 | 279/287 | 205/209/213 | 206/212 | 130 | 136/140/150 | 276/280/284 | 183/189 | 258/260/270 | 242/248 | 196 | 227 | 191/233 | 248 |
|  | 24A2 | 199/203 | 151/155 | 169/173 | 279/287 | 205/209/213 | 206/212 | 130 | 136/140/150 | 276/280/284 | 183/189 | 258/260/270 | 242/248 | 196 | 227 | 191/233 | 248 |
|  | 24A2 | 199/203 | 151/155 | 169/173 | 279/287 | 205/209/213 | 206/212 | 130 | 136/140/150 | 276/280/284 | 183/189 | 258/260/270 | 242/248 | 196 | 227 | 191/233 | 248 |
|  | 24A3 | 199/203 | 151/155 | 169/173 | 279/287 | 205/209/213 | 206/212 | 130 | 136/140/150 | 276/280/284 | 183/189 | 258/260/270 | 242/248 | 196 | 227 | 191/233 | 248 |
|  | 24A3 | 199/203 | 151/155 | 169/173 | 279/287 | 205/209/213 | 206/212 | 130 | 136/140/150 | 276/280/284 | 183/189 | 258/260/270 | 242/248 | 196 | 227 | 191/233 | 248 |
|  | 24B1 | 199/203 | 151/155 | 169/173 | 279/287 | 205/209/213 | 206/212 | 130 | 136/140/150 | 276/280/284 | 183/189 | 258/260/270 | 242/248 | 196 | 227 | 191/233 | 248 |
|  | 24B1 | 199/203 | 151/155 | 169/173 | 279/287 | 205/209/213 | 206/212 | 130 | 136/140/150 | 276/280/284 | 183/189 | 258/260/270 | 242/248 | 196 | 227 | 191/233 | 248 |
|  | 24B2 | 199/203 | 151/155 | 169/173 | 279/287 | 205/209/213 | 206/212 | 130 | 136/140/150 | 276/280/284 | 183/189 | 258/260/270 | 242/248 | 196 | 227 | 191/233 | 248 |
|  | 24B2 | 199/203 | 151/155 | 169/173 | 279/287 | 205/209/213 | 206/212 | 130 | 136/140/150 | 276/280/284 | 183/189 | 258/260/270 | 242/248 | 196 | 227 | 191/233 | 248 |
|  | 24B3 | 199/203 | 151/155 | 169/173 | 279/287 | 205/209/213 | 206/212 | 130 | 136/140/150 | 276/280/284 | 183/189 | 258/260/270 | 242/248 | 196 | 227 | 191/233 | 248 |
|  | 24B3 | 199/203 | 151/155 | 169/173 | 279/287 | 205/209/213 | 206/212 | 130 | 136/140/150 | 276/280/284 | 183/189 | 258/260/270 | 242/248 | 196 | 227 | 191/233 | 248 |
|  | 24C1 | 199/203 | 151/155 | 169/173 | -9 | 205/209/213 | 206/212 | 130 | 136/140/150 | 276/280/284 | 183/189 | 258/260/270 | 242/248 | 196 | 227 | 191/233 | 248 |
|  | 24C1 | 199/203 | 151/155 | 169/173 | 279/287 | 205/209/213 | 206/212 | 130 | 136/140/150 | 276/280/284 | 183/189 | 258/260/270 | 242/248 | 196 | 227 | 191/233 | 248 |
|  | 24C2 | 199/203 | 151/155 | 169/173 | 279/287 | 205/209/213 | 206/212 | 130 | 136/140/150 | 276/280/284 | 183/189 | 258/260/270 | 242/248 | 196 | 227 | 191/233 | 248 |
|  | 24C2 | 199/203 | 151/155 | 169/173 | 279/287 | 205/209/213 | 206/212 | 130 | 136/140/150 | 276/280/284 | 183/189 | 258/260/270 | 242/248 | 196 | 227 | 191/233 | 248 |
|  | 24C3 | 199/203 | 151/155 | 169/173 | 279/287 | 205/209/213 | 206/212 | 130 | 136/140/150 | 276/280/284 | 183/189 | 258/260/270 | 242/248 | 196 | 227 | 191/233 | 248 |
|  | 24C3 | 199/203 | 151/155 | 169/173 | 279/287 | 205/209/213 | 206/212 | 130 | 136/140/150 | 276/280/284 | 183/189 | 258/260/270 | 242/248 | 196 | 227 | 191/233 | 248 |
|  | 25M | 199/203 | 155 | 169/173 | 279/285 | 209/213 | 206 | 130 | 136/140/150 | 280/284 | 183/189 | 258/268 | 248 | 196/202 | 227 | 191 | 248 |
|  | 25A1 | 199/203 | 155 | 169/173 | 279/285 | 209/213 | 206 | 130 | 136/140/150 | 280/284 | 183/189 | 258/268 | 248 | 196/202 | 227 | 191 | 248 |
|  | 25A2 | 199/203 | 155 | 169/173 | 279/285 | 209/213 | 206 | 130 | 136/140/150 | 280/284 | 183/189 | 258/268 | 248 | 196/202 | 227 | 191 | 248 |
|  | 25A3 | 199/203 | 155 | 169/173 | 279/285 | 209/213 | 206 | 130 | 136/140/150 | 280/284 | 183/189 | 258/268 | 248 | 196/202 | 227 | 191 | 248 |
|  | 25B | 199/203 | 155 | -9 | -9 | 209/213 | 206 | 130 | -9 | -9 | 183/189 | 258 | -9 | 196/202 | 227 | -9 | -9 |
|  | 25B1 | 199/203 | 155 | 169/173 | 279/285 | 209/213 | 206 | 130 | 136/140/150 | 280/284 | 183/189 | 258/268 | 248 | 196/202 | 227 | 191 | 248 |
|  | 25B2 | 199/203 | 155 | 169/173 | 279/285 | 209/213 | 206 | 130 | 136/140/150 | 280/284 | 183/189 | 258/268 | 248 | 196/202 | 227 | 191 | 248 |
|  | 25B3 | 199/203 | 155 | 169/173 | 279/285 | 209/213 | 206 | 130 | 136/140/150 | 280/284 | 183/189 | 258/268 | 248 | 196/202 | 227 | 191 | 248 |
|  | 25C1 | 199/203 | 155 | 169/173 | 279/285 | 209/213 | 206 | 130 | 136/140/150 | 280/284 | 183/189 | 258/268 | 248 | 196/202 | 227 | 191 | 248 |
|  | 25C2 | 199/203 | 155 | 169/173 | 279/285 | 209/213 | 206 | 130 | 136/140/150 | 280/284 | 183/189 | 258/268 | 248 | 196/202 | 227 | 191 | 248 |
|  | 25C3 | 199/203 | 155 | 169/173 | 279/285 | 209/213 | 206 | 130 | 136/140/150 | 280/284 | 183/189 | 258/268 | 248 | 196/202 | 227 | 191 | 248 |
| g | 26M | 203/205 | 151/157 | 173 | 279/287 | 213/215 | 206 | 130 | 136/144/150 | 280/284 | 189 | 260 | 246 | 196 | 227 | 191/197 | 248 |
|  | 26A1 | 203/205 | 151/157 | 173 | 279/287 | 213/215 | 206 | 130 | 136/144/150 | 280/284 | 189 | 260 | 246 | 196 | 227 | 191/197 | 248 |
|  | 26A2 | 203/205 | 151/157 | 173 | 279/287 | 213/215 | 206 | 130 | 136/144/150 | 280/284 | 189 | 260 | 246 | 196 | 227 | 191/197 | 248 |
|  | 26A3 | 203/205 | 151/157 | 173 | 279/287 | 213/215 | 206 | 130 | 136/144/150 | 280/284 | 189 | 260 | 246 | 196 | 227 | 191/197 | 248 |
|  | 26B1 | 203/205 | 151/157 | 173 | 279/287 | 213/215 | 206 | 130 | 136/144/150 | 280/284 | 189 | 260 | 246 | 196 | 227 | 191/197 | 248 |
|  | 26B2 | 203/205 | 151/157 | 173 | 279/287 | 213/215 | 206 | 130 | 136/144/150 | 280/284 | 189 | 260 | 246 | 196 | 227 | 191/197 | 248 |
|  | 26B3 | 203/205 | 151/157 | 173 | 279/287 | 213/215 | 206 | 130 | 136/144/150 | 280/284 | 189 | 260 | 246 | 196 | 227 | 191/197 | 248 |
|  | 26C1 | 203/205 | 151/157 | 173 | 279/287 | 213/215 | 206 | 130 | 136/144/150 | 280/284 | 189 | 260 | 246 | 196 | 227 | 191/197 | 248 |
|  | 26C2 | 203/205 | 151/157 | 173 | 279/287 | 213/215 | 206 | 130 | 136/144/150 | 280/284 | 189 | 260 | 246 | 196 | 227 | 191/197 | 248 |
|  | 26C3 | 203/205 | 151/157 | 173 | 279/287 | 213/215 | 206 | 130 | 136/144/150 | 280/284 | 189 | 260 | 246 | 196 | 227 | 191/197 | 248 |
|  | 27M | 199/203 | 155 | 169/173 | 279/285 | 209/213 | 206 | 130 | 140/154 | 280/284 | 189 | 258/262/284 | 236/246 | 196 | 227 | -9 | 248 |
|  | 27A | 199/203 | 155 | 169/173 | 279/285 | 209/213 | 206 | 130 | 140/154 | 280/284 | 189 | 258/262/284 | 236/246 | 196 | 227 | -9 | 248 |
|  | 27B | 199/203 | 155 | 169/173 | 279/285 | 209/213 | 206 | 130 | 140/154 | 280/284 | 189 | 258/262/284 | 236/246 | 196 | 227 | -9 | 248 |
|  | 27C | 199/203 | 155 | 169/173 | 279/285 | 209/213 | 206 | 130 | 140/154 | 280/284 | 189 | 258/262/284 | 236/246 | 196 | 227 | -9 | 248 |
| h | 29M | 199/203 | 151/157 | 173 | 279/287 | 209/213 | 206 | 130 | 136/140/150 | 280/284 | 183/189 | 258 | 248 | 196 | 227 | 191/197 | 248 |
|  | 29A1 | 199/203 | 151/157 | 173 | 279/287 | 209/213 | 206 | 130 | 136/140/150 | 280/284 | 183/189 | 258 | 248 | 196 | 227 | 191/197 | 248 |
|  | 29A2 | 199/203 | 151/157 | 173 | 279/287 | 209/213 | 206 | 130 | 136/140/150 | 280/284 | 183/189 | 258 | 248 | 196 | 227 | 191/197 | 248 |
|  | 29A3 | 199/203 | 151/157 | 173 | 279/287 | 209/213 | 206 | 130 | 136/140/150 | 280/284 | 183/189 | 258 | 248 | 196 | 227 | 191/197 | 248 |
|  | 29B1 | 199/203 | 151/157 | 173 | 279/287 | 209/213 | 206 | 130 | 136/140/150 | 280/284 | 183/189 | 258 | 248 | 196 | 227 | 191/197 | 248 |
|  | 29B2 | 199/203 | 151/157 | 173 | 279/287 | 209/213 | 206 | 130 | 136/140/150 | 280/284 | 183/189 | 258 | 248 | 196 | 227 | 191/197 | 248 |
|  | 29B3 | 199/203 | 151/157 | 173 | 279/287 | 209/213 | 206 | 130 | 136/140/150 | 280/284 | 183/189 | 258 | 248 | 196 | 227 | 191/197 | 248 |
|  | 29C1 | 199/203 | 151/157 | 173 | 279/287 | 209/213 | 206 | 130 | 136/140/150 | 280/284 | 183/189 | 258 | 248 | 196 | 227 | 191/197 | 248 |
|  | 29C2 | 199/203 | 151/157 | 173 | 279/287 | 209/213 | 206 | 130 | 136/140/150 | 280/284 | 183/189 | 258 | 248 | 196 | 227 | 191/197 | 248 |
|  | 29C3 | 199/203 | 151/157 | 173 | 279/287 | 209/213 | 206 | 130 | 136/140/150 | 280/284 | 183/189 | 258 | 248 | 196 | 227 | 191/197 | 248 |
| i | 31M | 203 | -9 | -9 | -9 | 203/213/215 | 206 | 130 | 136/140/150 | 274/284 | 183/189 | 258 | 242/248 | 196/202 | 227 | -9 | 248 |
|  | 32M | 203 | -9 | -9 | -9 | 203/213/215 | 206 | 130 | 136/140/150 | 274/284 | 183/189 | 258 | 242/248 | 196/202 | 227 | -9 | 248 |
|  | 33M | 203 | -9 | -9 | -9 | 203/213/215 | 206 | 130 | 136/140/150 | 274/284 | 183/189 | 258 | 242/248 | 196/202 | 227 | -9 | 248 |
|  | 33A1 | 203 | -9 | -9 | -9 | 203/213/215 | 206 | 130 | 136/140/150 | 274/284 | 183/189 | 258 | 242/248 | 196/202 | 227 | -9 | 248 |
|  | 33A2 | 203 | -9 | -9 | -9 | 203/213/215 | 206 | 130 | 136/140/150 | 274/284 | 183/189 | 258 | 242/248 | 196/202 | 227 | -9 | 248 |
|  | 33A3 | 203 | -9 | -9 | -9 | 203/213/215 | 206 | 130 | 136/140/150 | 274/284 | 183/189 | 258 | 242/248 | 196/202 | 227 | -9 | 248 |
|  | 33B | 203 | -9 | -9 | -9 | 203/213/215 | 206 | 130 | 136/140/150 | 274/284 | 183/189 | 258 | 242/248 | 196/202 | 227 | -9 | 248 |
|  | 33C1 | 203 | -9 | -9 | -9 | 203/213/215 | 206 | 130 | 136/140/150 | 274/284 | 183/189 | 258 | 242/248 | 196/202 | 227 | -9 | 248 |
|  | 33C2 | 203 | -9 | -9 | -9 | 203/213/215 | 206 | 130 | 136/140/150 | 274/284 | 183/189 | 258 | 242/248 | 196/202 | 227 | -9 | 248 |
|  | 34M | 203 | -9 | -9 | -9 | 203/213/215 | 206 | 130 | 136/140/150 | 274/284 | 183/189 | 258 | 242/248 | 196/202 | 227 | -9 | 248 |
|  | 35M | 203 | -9 | -9 | -9 | 203/213/215 | 206 | 130 | 136/140/150 | 274/284 | 183/189 | 258 | 242/248 | 196/202 | 227 | -9 | 248 |
|  | 35A1 | 203 | -9 | -9 | -9 | 203/213/215 | 206 | 130 | 136/140/150 | 274/284 | 183/189 | 258 | 242/248 | 196/202 | 227 | -9 | 248 |
|  | 35A2 | 203 | -9 | -9 | -9 | 203/213/215 | 206 | 130 | 136/140/150 | 274/284 | 183/189 | 258 | 242/248 | 196/202 | 227 | -9 | 248 |
|  | 35A3 | 203 | -9 | -9 | -9 | 203/213/215 | 206 | 130 | 136/140/150 | 274/284 | 183/189 | 258 | 242/248 | 196/202 | 227 | -9 | 248 |
|  | 35B1 | 203 | -9 | -9 | -9 | 203/213/215 | 206 | 130 | 136/140/150 | 274/284 | 183/189 | 258 | 242/248 | 196/202 | 227 | -9 | 248 |
|  | 35B2 | 203 | -9 | -9 | -9 | 203/213/215 | 206 | 130 | 136/140/150 | 274/284 | 183/189 | 258 | 242/248 | 196/202 | 227 | -9 | 248 |
|  | 35B3 | 203 | -9 | -9 | -9 | 203/213/215 | 206 | 130 | 136/140/150 | 274/284 | 183/189 | 258 | 242/248 | 196/202 | 227 | -9 | 248 |
|  | 35C1 | 203 | -9 | -9 | -9 | 203/213/215 | 206 | 130 | 136/140/150 | 274/284 | 183/189 | 258 | 242/248 | 196/202 | 227 | -9 | 248 |
|  | 35C2 | 203 | -9 | -9 | -9 | 203/213/215 | 206 | 130 | 136/140/150 | 274/284 | 183/189 | 258 | 242/248 | 196/202 | 227 | -9 | 248 |
|  | 38M | 203 | -9 | -9 | -9 | 203/213/215 | 206 | 130 | 136/140/150 | 274/284 | 183/189 | 258 | 242/248 | 196/202 | 227 | -9 | 248 |
|  | 38A1 | 203 | -9 | -9 | -9 | 203/213/215 | 206 | 130 | 136/140/150 | 274/284 | 183/189 | 258 | 242/248 | 196/202 | 227 | -9 | 248 |
|  | 38A2 | 203 | -9 | -9 | -9 | 203/213/215 | 206 | 130 | 136/140/150 | 274/284 | 183/189 | 258 | 242/248 | 196/202 | 227 | -9 | 248 |
|  | 38B1 | 203 | -9 | -9 | -9 | 203/213/215 | 206 | 130 | 136/140/150 | 274/284 | 183/189 | 258 | 242/248 | 196/202 | 227 | -9 | 248 |
|  | 38B2 | 203 | -9 | -9 | -9 | 203/213/215 | 206 | 130 | 136/140/150 | 274/284 | 183/189 | 258 | 242/248 | 196/202 | 227 | -9 | 248 |
|  | 38C1 | 203 | -9 | -9 | -9 | 203/213/215 | 206 | 130 | 136/140/150 | 274/284 | 183/189 | 258 | 242/248 | 196/202 | 227 | -9 | 248 |
|  | 38C2 | 203 | -9 | -9 | -9 | 203/213/215 | 206 | 130 | 136/140/150 | 274/284 | 183/189 | 258 | 242/248 | 196/202 | 227 | -9 | 248 |
| j | 44M | 203 | 151/155 | 169/173 | 279/287 | 209/213 | 206 | 130 | 136/140/150 | 280/284 | 177/183/189 | 256/264/266 | 246 | 196 | 227 | -9 | 248 |
|  | 44-1 | 203 | 151/155 | 169/173 | 279/287 | 209/213 | 206 | 130 | 136/140/150 | 280/284 | 177/183/189 | 256/264/266 | 246 | 196 | 227 | -9 | 248 |
|  | 44-2 | 203 | 151/155 | 169/173 | 279/287 | 209/213 | 206 | 130 | 136/140/150 | 280/284 | 177/183/189 | 256/264/266 | 246 | 196 | 227 | -9 | 248 |
|  | 44-3 | 203 | 151/155 | 169/173 | 279/287 | 209/213 | 206 | 130 | 136/140/150 | 280/284 | 177/183/189 | 256/264/266 | 246 | 196 | 227 | -9 | 248 |
|  | 45M | 203 | 151/155 | 169/173 | 279/287 | 209/213 | 206 | 130 | 136/140/150 | 280/284 | 177/183/189 | 256/264/266 | 246 | 196 | 227 | -9 | 248 |
|  | 45-1 | 203 | 151/155 | 169/173 | 279/287 | 209/213 | 206 | 130 | 136/140/150 | 280/284 | 177/183/189 | 256/264/266 | 246 | 196 | 227 | -9 | 248 |
|  | 45-2 | 203 | 151/155 | 169/173 | 279/287 | 209/213 | 206 | 130 | 136/140/150 | 280/284 | 177/183/189 | 256/264/266 | 246 | 196 | 227 | -9 | 248 |
|  | 45-3 | 203 | 151/155 | 169/173 | 279/287 | 209/213 | 206 | 130 | 136/140/150 | 280/284 | 177/183/189 | 256/264/266 | 246 | 196 | 227 | -9 | 248 |
|  | 45-4 | 203 | 151/155 | 169/173 | 279/287 | 209/213 | 206 | 130 | 136/140/150 | 280/284 | 177/183/189 | 256/264/266 | 246 | 196 | 227 | -9 | 248 |
|  | 45-5 | 203 | 151/155 | 169/173 | 279/287 | 209/213 | 206 | 130 | 136/140/150 | 280/284 | 177/183/189 | 256/264/266 | 246 | 196 | 227 | -9 | 248 |
|  | 45-6 | 203 | 151/155 | 169/173 | 279/287 | 209/213 | 206 | 130 | 136/140/150 | 280/284 | 177/183/189 | 256/264/266 | 246 | 196 | 227 | -9 | 248 |
|  | 45-7 | 203 | 151/155 | 169/173 | 279/287 | 209/213 | 206 | 130 | 136/140/150 | 280/284 | 177/183/189 | 256/264/266 | 246 | 196 | 227 | -9 | 248 |
|  | 45-8 | 203 | 151/155 | 169/173 | 279/287 | 209/213 | 206 | 130 | 136/140/150 | 280/284 | 177/183/189 | 256/264/266 | 246 | 196 | 227 | -9 | 248 |
|  | 46M | 205 | 151/157 | 175 | 281/283 | 209/213 | 206 | 130/138 | 136/140/150 | 280/284 | 189 | 258/262 | 246/248 | 196 | 227 | 209/217 | 248 |
|  | 46M | 205 | 151/157 | 175 | 281/283 | 209/213 | 206 | 130/138 | 136/140/150 | 280/284 | 189 | 262 | 248 | 196 | 227 | 209/217 | 248 |
|  | 46A1 | 205 | 151/157 | 175 | 281/283 | 209/213 | 206 | 130/138 | 136/140/150 | 280/284 | 189 | 258/262 | 246/248 | 196 | 227 | 209/217 | 248 |
|  | 46B1 | 205 | 151/157 | 175 | 281/283 | 209/213 | 206 | 130/138 | 136/140/150 | 280/284 | 189 | 258/262 | 246/248 | 196 | 227 | 209/217 | 248 |
|  | 46B2 | 205 | 151/157 | 175 | 281/283 | 209/213 | 206 | 130/138 | 136/140/150 | 280/284 | 189 | 258/262 | 246/248 | 196 | 227 | 209/217 | 248 |
|  | 46B3 | 205 | 151/157 | 175 | 281/283 | 209/213 | 206 | 130/138 | 136/140/150 | 280/284 | 189 | 258/262 | 246/248 | 196 | 227 | 209/217 | 248 |
|  | 46C1 | 205 | 151/157 | 175 | 281/283 | 209/213 | 206 | 130/138 | 136/140/150 | 280/284 | 189 | 258/262 | 246/248 | 196 | 227 | 209/217 | 248 |
|  | 46C2 | 205 | 151/157 | 175 | 281/283 | 209/213 | 206 | 130/138 | 136/140/150 | 280/284 | 189 | 258/262 | 246/248 | 196 | 227 | 209/217 | 248 |
|  | 46C3 | 205 | 151/157 | 175 | 281/283 | 209/213 | 206 | 130/138 | 136/140/150 | 280/284 | 189 | 258/262 | 246/248 | 196 | 227 | 209/217 | 248 |
|  | 47M | 205 | 151/157 | 175 | 281/283 | 209/213 | 206 | 130/138 | 136/140/150 | 280/284 | 189 | 258/262 | 246/248 | 196 | 227 | 209/217 | 248 |
|  | 47M | 205 | 151/157 | 175 | 281/283 | 209/213 | 206 | 130/138 | 136/140/150 | 280/284 | 189 | 258/262 | 248 | 196 | 227 | 209/217 | 248 |
|  | 47A1 | 205 | 151/157 | 175 | 281/283 | 209/213 | 206 | 130/138 | 136/140/150 | 280/284 | 189 | 258/262 | 246/248 | 196 | 227 | 209/217 | 248 |
|  | 47A2 | 205 | 151/157 | 175 | 281/283 | 209/213 | 206 | 130/138 | 136/140/150 | 280/284 | 189 | 258/262 | 246/248 | 196 | 227 | 209/217 | 248 |
|  | 47A3 | 205 | 151/157 | 175 | 281/283 | 209/213 | 206 | 130/138 | 136/140/150 | 280/284 | 189 | 258/262 | 246/248 | 196 | 227 | 209/217 | 248 |
|  | 47B1 | 205 | 151/157 | 175 | 281/283 | 209/213 | 206 | 130/138 | 136/140/150 | 280/284 | 189 | 258/262 | 246/248 | 196 | 227 | 209/217 | 248 |
|  | 47B2 | 205 | 151/157 | 175 | 281/283 | 209/213 | 206 | 130/138 | 136/140/150 | 280/284 | 189 | 258/262 | 246/248 | 196 | 227 | 209/217 | 248 |
|  | 47B3 | 205 | 151/157 | 175 | 281/283 | 209/213 | 206 | 130/138 | 136/140/150 | 280/284 | 189 | 258/262 | 246/248 | 196 | 227 | 209/217 | 248 |
|  | 47C1 | 205 | 151/157 | 175 | 281/283 | 209/213 | 206 | 130/138 | 136/140/150 | 280/284 | 189 | 258/262 | 246/248 | 196 | 227 | 209/217 | 248 |
|  | 47C2 | 205 | 151/157 | 175 | 281/283 | 209/213 | 206 | 130/138 | 136/140/150 | 280/284 | 189 | 258/262 | 246/248 | 196 | 227 | 209/217 | 248 |
|  | 47C3 | 205 | 151/157 | 175 | 281/283 | 209/213 | 206 | 130/138 | 136/140/150 | 280/284 | 189 | 258/262 | 246/248 | 196 | 227 | 209/217 | 248 |
| k | 40M | 187/199 | -9 | -9 | -9 | 215/217 | 206 | 130 | 130/136/140/148 | 280/288 | 189 | 248/252/260/270 | 242/248 | 194/196/202/206 | 227 | -9 | 248 |
|  | 40-1 | 187/199 | -9 | -9 | -9 | 215/217 | 206 | 130 | 130/136/140/148 | 280/288 | 189 | 248/252/260/270 | 242/248 | 194/196/202/206 | 227 | -9 | 248 |
|  | 40-2 | 187/199 | -9 | -9 | -9 | 215/217 | 206 | 130 | 130/136/140/148 | 280/288 | 189 | 248/252/260/270 | 242/248 | 194/196/202/206 | 227 | -9 | 248 |
|  | 40-3 | 187/199 | -9 | -9 | -9 | 215/217 | 206 | 130 | 130/136/140/148 | 280/288 | 189 | 248/252/260/270 | 242/248 | 194/196/202/206 | 227 | -9 | 248 |
| l | EM1m | 203 | -9 | 173 | 281/287 | 205 | 206/212 | 130 | 136 | 276/284/286 | 183/189 | 258 | 238/246/250 | 196/202 | 227 | 191/197/205 | 248 |
|  | EM1 | 203 | -9 | -9 | -9 | 203/213 | 206 | 130 | 136/140/150 | 280/284 | 183/189 | 258 | 246 | 196/202 | 227 | -9 | 248 |
|  | EM1-1 | 203 | -9 | 173 | 281/287 | 205 | 206/212 | 130 | 136 | 276/284/286 | 183/189 | 258 | 238/246/250 | 196/202 | 227 | 191/197/205 | 248 |
|  | EM1-2 | 203 | -9 | 173 | 281/287 | 205 | 206/212 | 130 | 136 | 276/284/286 | 183/189 | 258 | 238/246/250 | 196/202 | 227 | 191/197/205 | 248 |
|  | EM1-3 | 203 | -9 | 173 | -9 | 205 | 206/212 | 130 | 136 | 276/284/286 | 183/189 | 258 | -9 | 196/202 | 227 | 191/197/205 | 248 |
|  | EM2m | 203 | -9 | 173 | -9 | 203/213 | 206 | 130 | 140 | 274/284 | 183/189 | 258 | 242/246 | 196/202 | 227 | 197/205/219 | 248 |
|  | EM2 | 203 | -9 | -9 | -9 | 203/213 | 206 | 130 | 136/140/150 | 280/284 | 183/189 | 258 | 246 | 196/202 | 227 | -9 | 248 |
|  | EM2-1 | 203 | 149/153/155 | 173 | -9 | 203/213 | 206 | 130 | 140 | 274/284 | 183/189 | 258 | 242/246 | 196/202 | 227 | 197/205/219 | 248 |
|  | EM2-2 | 203 | 149/153/155 | 173 | -9 | 203/213 | 206 | 130 | 140 | 274/284 | 183/189 | 258 | -9 | 196/202 | 227 | 197/205/219 | 248 |
|  | EM2-3 | 203 | 149/153/155 | 173 | -9 | 203/213 | 206 | 130 | 140 | 274/284 | 183/189 | 258 | -9 | 196/202 | 227 | 197/205/219 | 248 |
|  | EM3m | 203 | 149/153/155 | 173 | -9 | 203/213 | 206 | 130 | 140 | 274/284 | 183/189 | 258 | 242/246 | 196/202 | 227 | 197/205/219 | 248 |
|  | EM3 | 203 | -9 | -9 | -9 | 203/213 | 206 | 130 | 136/150 | 280/284 | 177/183/189 | 256/264/266 | 246 | 196 | 227 | -9 | 248 |
|  | EM3-1 | 203 | 149/153/155 | 173 | -9 | 203/213 | 206 | 130 | 140 | 274/284 | 183/189 | 258 | 242/246 | 196/202 | 227 | 197/205/219 | 248 |
|  | EM3-2 | 203 | 149/153/155 | 173 | -9 | 205 | 206/212 | 130 | 136 | -9 | -9 | -9 | -9 | 196/202 | 227 | 191/197/205 | 248 |
|  | EM3-3 | 203 | 149/153/155 | 173 | -9 | 203/213 | 206 | 130 | 140 | 274/284 | 183/189 | 258 | 242/246 | 196/202 | 227 | 197/205/219 | 248 |
|  | EM4m | 203 | 151 | 163/173 | -9 | 203/213 | 206 | 130 | 140 | 274/284 | 183/189 | 258 | 242/246 | 196/202 | 227 | 197/205/219 | 248 |
|  | EM4-1 | 203 | 151 | 163/173 | -9 | 203/213 | 206 | 130 | 140 | 274/284 | 183/189 | 258 | 242/246 | 196/202 | 227 | 197/205/219 | 248 |
|  | EM4-2 | 203 | 151 | 163/173 | -9 | 203/213 | 206 | 130 | 140 | 274/284 | 183/189 | 258 | -9 | 196/202 | 227 | 197/205/219 | 248 |
|  | EM4-3 | 203 | 151 | 163/173 | -9 | 203/213 | 206 | 130 | 140 | 274/284 | 183/189 | 258 | -9 | 196/202 | 227 | 197/205/219 | 248 |
|  | EM6m | 203 | 143/149/153 | 163/173 | -9 | 205 | 206/212 | 130 | 136 | 276/284/286 | 183/189 | 258 | 238/246/250 | 196 | 227 | 191/197/205 | 248 |
|  | EM6-1a | 203 | 143/149/153 | 163/173 | 275/283/287 | 205 | 206/212 | 130 | 136 | 276/284/286 | 183/189 | 258 | -9 | 196 | 227 | 191/197/205 | 248 |
|  | EM6-1b | 203 | 143/149/153 | 163/173 | 275/283/287 | 205 | 206/212 | 130 | 136 | 276/284/286 | 183/189 | 258 | -9 | 196 | 227 | 191/197/205 | 248 |
|  | EM6-1c | 203 | 143/149/153 | 163/173 | 275/283/287 | 205 | 206/212 | 130 | 136 | 276/284/286 | 183/189 | 258 | -9 | 196 | 227 | 191/197/205 | 248 |
|  | EM6-2a | 203 | 143/149/153 | 163/173 | 275/283/287 | 205 | 206/212 | 130 | 136 | 276/284/286 | 183/189 | 258 | -9 | 196 | 227 | 191/197/205 | 248 |
|  | EM6-2b | 203 | 143/149/153 | 163/173 | -9 | 205 | 206/212 | 130 | 136 | 276/284/286 | 183/189 | 258 | -9 | 196 | 227 | 191/197/205 | 248 |
|  | EM6-3b | 203 | 143/149/153 | 163/173 | 275/283/287 | 205 | 206/212 | 130 | 136 | 276/284/286 | 183/189 | 258 | -9 | 196 | 227 | 191/197/205 | 248 |
|  | EM6-4a | 203 | 143/149/153 | 163/173 | 275/283/287 | 205 | 206/212 | 130 | 136 | 276/284/286 | 183/189 | 258 | 238/246/250 | 196 | 227 | 191/197/205 | 248 |
|  | EM7m | -9 | 153 | 173 | -9 | 203/213 | 206 | 130 | 140 | 274/284 | 183/189 | 258 | -9 | 196/202 | 227 | 197/205/219 | 248 |
|  | EM7-1a | 203 | 153 | 173 | -9 | 203/213 | 206 | 130 | 140 | 274/284 | 183/189 | 258 | -9 | 196/202 | 227 | 197/205/219 | 248 |
|  | EM7-1b | -9 | -9 | 173 | -9 | 203/213 | 206 | 130 | 140 | 274/284 | 183/189 | 258 | -9 | 196/202 | 227 | 197/205/219 | 248 |
|  | EM7-1c | -9 | -9 | 173 | -9 | 203/213 | 206 | 130 | 140 | 274/284 | 183/189 | 258 | -9 | 196/202 | 227 | 197/205/219 | 248 |
|  | EM7-2a | 203 | -9 | 173 | -9 | 203/213 | 206 | 130 | 140 | 274/284 | 183/189 | 258 | -9 | 196/202 | 227 | 197/205/219 | 248 |
|  | EM7-2b | -9 | -9 | 173 | -9 | 203/213 | 206 | 130 | 140 | 274/284 | 183/189 | 258 | -9 | 196/202 | 227 | 197/205/219 | 248 |
|  | EM7-3a | 203 | -9 | 173 | -9 | 203/213 | 206 | 130 | 140 | 274/284 | 183/189 | 258 | -9 | 196/202 | 227 | 197/205/219 | 248 |
|  | EM7-3b | -9 | -9 | 173 | -9 | 203/213 | 206 | 130 | 140 | 274/284 | 183/189 | 258 | -9 | 196/202 | 227 | 197/205/219 | 248 |
|  | EM7-3c | -9 | -9 | 173 | -9 | 203/213 | 206 | 130 | 140 | 274/284 | 183/189 | 258 | -9 | 196/202 | 227 | 197/205/219 | 248 |
|  | EM8m | -9 | 151 | 173 | -9 | 203/213 | 206 | 130 | 140 | 274/284 | 183/189 | 258 | 242/246 | 196/202 | 227 | 197/205/219 | 248 |
|  | EM8-1a | -9 | -9 | 173 | -9 | 203/213 | 206 | 130 | 140 | 274/284 | 183/189 | 258 | -9 | 196/202 | 227 | 197/205/219 | 248 |
|  | EM8-1b | -9 | -9 | 173 | -9 | 203/213 | 206 | 130 | 140 | 274/284 | 183/189 | 258 | -9 | 196/202 | 227 | 197/205/219 | 248 |
|  | EM8-1c | -9 | -9 | 173 | -9 | 203/213 | 206 | 130 | 140 | 274/284 | 183/189 | 258 | -9 | 196/202 | 227 | 197/205/219 | 248 |
|  | EM8-2a | 203 | -9 | 173 | -9 | 203/213 | 206 | 130 | 140 | 274/284 | 183/189 | 258 | -9 | 196/202 | 227 | 197/205/219 | 248 |
|  | EM8-2b | 203 | -9 | 173 | -9 | 203/213 | 206 | 130 | 140 | 274/284 | 183/189 | 258 | -9 | 196/202 | 227 | 197/205/219 | 248 |
|  | EM8-2c | 203 | -9 | 173 | -9 | 203/213 | 206 | 130 | 140 | 274/284 | 183/189 | 258 | -9 | 196/202 | 227 | 197/205/219 | 248 |
|  | EM10 | 203 | -9 | 173 | -9 | 203/213 | 206 | 130 | 140 | 274/284 | 183/189 | 258 | -9 | 196/202 | 227 | 197/205/219 | 248 |
|  | EM10-1 | 203 | -9 | 173 | -9 | 203/213 | 206 | 130 | 140 | 274/284 | 183/189 | 258 | -9 | 196/202 | 227 | 197/205/219 | 248 |
|  | EM10-2 | 203 | -9 | 173 | -9 | 203/213 | 206 | 130 | 140 | 274/284 | 183/189 | 258 | -9 | 196/202 | 227 | 197/205/219 | 248 |
|  | EM10-3a | 203 | -9 | 173 | -9 | 203/213 | 206 | 130 | 140 | 274/284 | 183/189 | 258 | -9 | 196/202 | 227 | 197/205/219 | 248 |
|  | EM10-3b | 203 | -9 | 173 | -9 | 203/213 | 206 | 130 | 140 | 274/284 | 183/189 | 258 | -9 | 196/202 | 227 | 197/205/219 | 248 |
|  | EM11m | -9 | 153 | 173 | -9 | 203/213 | 206 | 130 | 140 | 274/284 | 183/189 | 258 | -9 | 196/202 | 227 | 197/205/219 | 248 |
| m | ZR1-1 | -9 | -9 | 173 | -9 | 209 | 206 | 130/138 | 136/150 | 280 | 189 | 260 | -9 | 196 | 227 | 191/211 | 248 |
|  | ZR1-2 | -9 | -9 | -9 | -9 | 213 | 206 | 130/138 | 136/138 | 280/284 | 177 | 256/260 | 246/248 | 196 | 227 | 189 | 248 |
|  | ZR1-3 | 199/203 | -9 | 169/173 | -9 | 209 | 206 | -9 | 136 | 280/284 | 183/189 | 258/260 | 242/248 | 196 | 227 | 191 | 248 |
|  | ZR1-4 | 199/203 | -9 | 169/173 | 281 | 213 | 206 | -9 | 136 | 284/292 | 177/183 | 258/274 | -9 | 196 | 227 | 189/199 | 248 |
|  | ZR1-5 | -9 | -9 | 173 | -9 | 213 | 206/212 | -9 | 136/138 | 284 | 189 | 258 | 248 | 196 | 227 | 191/197 | 248 |
|  | ZR1-6 | -9 | -9 | -9 | -9 | 213 | 206/212 | -9 | 138 | 284 | 183/189 | 258 | 246/248 | 196 | 227 | 191/215 | 248 |
|  | ZR1-7 | 189/203 | -9 | 159/173 | -9 | 209/215 | 206 | -9 | 136 | 280/286 | 183/189 | 258 | -9 | 196 | 227 | 187/191 | 244/248 |
|  | ZR1-8 | 199 | -9 | 169 | -9 | 209 | 206 | -9 | 136/150 | 280/284 | 177/189 | 268 | 248 | 196 | 227 | 191 | 248 |
|  | ZR1-9 | -9 | -9 | -9 | -9 | 213 | 206 | -9 | 140 | 284 | 183/189 | 258 | -9 | 196 | 227 | 187/203 | 248 |
|  | ZR1-10 | -9 | -9 | -9 | -9 | 203/215 | 206 | -9 | 136/138 | -9 | 183/189 | 258 | -9 | 196 | 227 | 187/197 | 248 |
|  | ZR1-11 | 203 | -9 | 173 | 281 | 215 | 206 | -9 | 136/138 | 286 | 177/189 | 258 | 248/250 | 196 | 227 | 187/197 | 248 |
|  | ZR1-12 | 203/205 | -9 | 173 | -9 | 213 | 206 | -9 | 144/150 | 284 | 177 | 258/262 | 246/248 | 196/202 | 227 | 191/217 | 248 |
|  | ZR1-13 | -9 | -9 | 159/173 | -9 | 213 | 206 | -9 | 140/144 | 278/284 | 183/189 | 260 | -9 | 196 | 227 | 187 | 248 |
|  | ZR1-14 | -9 | -9 | 173 | -9 | 215 | 206 | -9 | 136/138 | 286 | 177/189 | 258 | -9 | 196 | 227 | 187/197 | 248 |
|  | ZR1-15 | -9 | -9 | 173 | -9 | 213 | 206 | -9 | 144/150 | 284 | 177 | 258/262 | -9 | 196 | 227 | 191/217 | 248 |
|  | ZR1-16 | -9 | -9 | -9 | -9 | 209/213 | 206 | -9 | 136 | 280/284 | 183/189 | 266/270 | -9 | 196 | 227 | 187/191 | 248 |
|  | ZR1-17 | -9 | -9 | -9 | -9 | 209/215 | 206 | -9 | 136 | -9 | 177/181 | 262/266 | -9 | 196 | 227 | 187/191 | 248 |
|  | ZR1-18 | -9 | -9 | 173 | -9 | 209 | 206/212 | -9 | 136 | 280/284 | 189 | 268/274 | -9 | 196/202 | 227 | 199/209 | 248 |
|  | ZR1-19 | -9 | -9 | 169/173 | -9 | 209 | 206 | -9 | 144 | 280/284 | 183 | 254 | -9 | 196 | 227 | 197 | 244/248 |
|  | ZR1-20 | 203/205 | -9 | -9 | -9 | 209/213 | 206 | -9 | 136 | 284 | 183/189 | 252 | -9 | 196 | 227 | 187/191 | 248 |
| n | ZR2-1 | -9 | -9 | -9 | -9 | 209 | 206 | -9 | 136/140 | 280 | 177/183 | 252/258 | -9 | 196 | 227 | 191 | 248 |
|  | ZR2-2 | 203 | -9 | 173 | 281/283 | 205 | 206 | -9 | 136/148 | 284 | 183/189 | 254 | -9 | 196 | 225/227 | 191/199 | 248 |
|  | ZR2-3 | -9 | -9 | -9 | -9 | 209 | 206/212 | -9 | 136 | 280 | 189 | 256/268 | -9 | 196/202 | 227 | 191/211 | 248 |
|  | ZR2-4 | -9 | -9 | 173 | -9 | 209 | 206/212 | -9 | 136/148 | 280/284 | 183/189 | 260 | -9 | 196 | 227 | 191 | 248 |
|  | ZR2-5 | -9 | -9 | -9 | -9 | 213 | 212 | 130 | 136 | 278 | 177/183 | 260/270 | 248/250 | 196/202 | 227 | 197/201 | 248 |
|  | ZR2-6 | -9 | -9 | 173 | -9 | 209/213 | 206/212 | 130 | 136 | 278/284 | 183 | 260 | 248 | 196/202 | 227 | 270 | 248 |
| o | BJ1-1 | -9 | -9 | -9 | -9 | 213/215 | 206 | 130/138 | 140 | 284 | 189 | 262/276 | - | 196/202 | 227 | 187/191 | 248 |
|  | BJ1-2 | -9 | -9 | -9 | -9 | 213/215 | 206 | 130/138 | 140 | - | 189 | 262/276 | 236/246 | 196/202 | 227 | 187/191 | 248 |
|  | BJ1-3 | 199/203 | -9 | -9 | -9 | 209 | 206 | 130/138 | 136 | 280 | 177/189 | 262/268 | 248/250 | 196 | 227/233 | 191 | 248 |
|  | BJ1-4 | -9 | -9 | -9 | -9 | 213/215 | 206 | 130 | 140 | 284 | 189 | 262/276 | 236/246 | 196 | 227 | 187/191 | 248 |
|  | BJ1-5 | 203 | -9 | -9 | -9 | 209 | 206 | 138 | 136 | 280 | 189 | 256/268 | - | 196 | 227 | 191/225 | 248 |
|  | BJ1-6 | -9 | -9 | 169/173 | -9 | 213 | 206/212 | 130 | 136/140 | - | 177/189 | 256/262 | -9 | 192/196 | 227 | 191 | 248 |
|  | BJ1-7 | 199 | -9 | -9 | -9 | 209 | 206 | 130 | 136 | - | 177/189 | 262/268 | 248/250 | 192/196 | 227/233 | 191/215 | 248 |
|  | BJ1-8 | -9 | -9 | -9 | -9 | 215 | 206 | 130 | 136/150 | 284 | 189 | 260 | 242 | 192/196 | 227 | 187/191 | 248 |
|  | BJ1-9 | 189/203 | -9 | -9 | -9 | 213/215 | 206 | 130 | 136/144 | - | 183/189 | 266/268 | 248 | 192/196 | 227 | 207 | 248 |
|  | BJ1-10 | -9 | -9 | -9 | -9 | 213 | 206 | 130 | 136/144 | 284 | 183/189 | 258/282 | 248 | 192/196 | 227 | 191/213 | 244/248 |
|  | BJ1-11 | -9 | -9 | -9 | -9 | 209 | 206/212 | 130 | 140 | 280/284 | 183/189 | 256/260 | 248 | 196 | 227 | 191/209 | 248 |
|  | BJ1-12 | -9 | -9 | -9 | -9 | 209 | 206/212 | 130 | 144/150 | 280 | 177/189 | 256/282 | 248 | 196/202 | 227 | 191 | 248 |
|  | BJ1-13 | 199 | -9 | -9 | -9 | 213 | 206/212 | 130 | 140 | 284 | 189 | 256/266 | - | 196/202 | 227 | 191/229 | 248 |
|  | BJ1-14 | -9 | -9 | 173 | -9 | 209/213 | 206 | 130/138 | 144/158 | 280/284 | 177/189 | 256/258 | -9 | 196/202 | 227 | 191/209 | 248 |
|  | BJ1-15 | -9 | -9 | -9 | -9 | 213/215 | 212 | 130/138 | 138/152 | - | 177/183 | 256/272 | 246/248 | 196 | 227 | 203 | 248 |
|  | BJ1-16 | 203/205 | -9 | -9 | -9 | 209/213 | 206 | 130 | 144 | 284 | 177/189 | 246/256 | 226/248 | 196/202 | 223/227 | 191 | 244/248 |
|  | BJ1-17 | -9 | -9 | -9 | -9 | 213 | 206 | 130/138 | 136 | 284 | 177/189 | 256/262 | -9 | 196/202 | 227 | 191/215 | 244/248 |
|  | BJ1-18 | -9 | -9 | -9 | -9 | 209 | 206/212 | 130 | 136 | - | 177/189 | 256/260 | -9 | 196/202 | 227 | 201/209 | 248 |
|  | BJ1-19 | -9 | -9 | -9 | -9 | 209 | 206/212 | 130 | 136/140 | - | 177 | 256/262 | -9 | 196/202 | 227 | 191/197 | 248 |
|  | BJ1-20 | 203 | -9 | -9 | -9 | 213 | 206/212 | 130/138 | 136 | 284 | 177/191 | 256/262 | 242/248 | 196/202 | 227 | 191/225 | 248 |
|  | BJ1-22 | -9 | -9 | 173 | -9 | 205/209 | 206 | 130 | 136/150 | 276/280 | 177/191 | 260/266 | 246/248 | 196 | 227 | 191/225 | 248 |
|  | BJ1-23 | -9 | -9 | -9 | -9 | 209/213 | 206 | 130 | 136/144 | 284 | 183/189 | 266/268 | -9 | 196 | 227 | 207/239 | 248 |
|  | BJ1-24 | -9 | -9 | -9 | -9 | 209 | 206/212 | 130 | 136 | - | 177/189 | 256/260 | 248 | 196 | 227 | 201/209 | 248 |
|  | BJ1-25 | -9 | -9 | -9 | -9 | 209/213 | 206 | 130 | 136/144 | 284 | 183/189 | 266/268 | 242/248 | 196 | 227 | 207/239 | 248 |
|  | BJ1-26 | -9 | -9 | -9 | -9 | 209/213 | 206/212 | 130 | 136/140 | - | 177/189 | 252/262 | 246/248 | 196 | 227 | 201/231 | 248 |
| p | BJ2-1 | -9 | -9 | -9 | -9 | 209/215 | 206 | 130 | 150/154 | 280/286 | 183/189 | 250/268 | 248/250 | 196/202 | 227 | 191/203 | 244/248 |
|  | BJ2-2 | -9 | -9 | -9 | -9 | 209/215 | 206 | 130/138 | 136/140 | - | 183/189 | 250 | - | 196/202 | 227 | 191/203 | 248 |
|  | BJ2-3 | 199/203 | -9 | -9 | -9 | 209/215 | 206 | 130 | 150/154 | 280/284 | 183/189 | 250/268 | 248/250 | 196 | 227 | 191/203 | 244/248 |
|  | BJ2-4 | -9 | -9 | -9 | -9 | 209 | 212 | 130 | 136/144 | 280 | 177/189 | 252/266 | 248/250 | 196/202 | 227 | 219/231 | 248 |
|  | BJ2-5 | -9 | -9 | 169/173 | -9 | 209 | 212 | 130 | 136/144 | 280 | 177/189 | 252/266 | 248/250 | 196/202 | 227 | 219/231 | 248 |
| CK | | -9 | -9 | -9 | -9 | -9 | -9 | -9 | -9 | -9 | -9 | -9 | -9 | -9 | -9 | -9 | -9 |

Note: **P-ID**, population ID. **SG**, sample groups.

Table S9 Ploidy estimated by SSR maximum allele number and ploidy detected by FCM

| **population ID** | **SSR samples (N)** | **maximum number alleles** | **predictive ploidy (2n)** | **FCM samples (N)** | **FCM ploidy test value** | **ploidy level (2n)** | **reproduction mode** |
| --- | --- | --- | --- | --- | --- | --- | --- |
| **a** | 5 | 3 | 3X | 2 | 3.45-3.48 | 3X | Apomictic |
| **b** | 10 | 3 | 3X | 4 | 3.19-3.49 | 3X | Apomictic |
| **c** | 47 | 2 | 2X | 16 | 2.05-2.47 | 2X | Sexual |
| **d** | 4 | 3 | 3X | - | - | - | Apomictic |
| **e** | 10 | 3 | 3X | 1 | 3.26 | 3X | Apomictic |
| **f** | 24 | 3 | 3X | 8 | 2.79-3.43 | 3X | Apomictic |
| **g** | 14 | 3 | 3X | 4 | 3.27-3.50 | 3X | Apomictic |
| **h** | 10 | 3 | 3X | 4 | 3.27-3.50 | 3X | Apomictic |
| **i** | 26 | 3 | 3X | 14 | 2.91-3.38 | 3X | Apomictic |
| **j** | 31 | 3 | 3X | 12 | 2.78-3.55 | 3X | Apomictic |
| **k** | 4 | 4 | 4X | 3 | 4.00-4.27 | 4X | Apomictic |
| **l** | 46 | 3 | 3X | 14 | 2.93-3.51 | 3X | Apomictic |
| **m** | 20 | 2 | 2X | 3 | 2.17-2.39 | 2X | Sexual |
| **n** | 6 | 2 | 2X | 2 | 2.39-2.42 | 2X | Sexual |
| **o** | 25 | 2 | 2X | 3 | 2.15-2.19 | 2X | Sexual |
| **p** | 5 | 2 | 2X | 2 | 2.09-2.41 | 2X | Sexual |
