# Supplementary material for: Polyembryonic or Apomictic Seeds Enable Fig Trees to Establish New Populations Without Their Pollinating Wasps, a Case Observation in Ficus gasparriniana
Source: Ecol Evol. 2025 Oct 16;15(10):e72316. doi: 10.1002/ece3.72316 (PMC12528958; doi:10.1002/ece3.72316)
Supplement: Supplementary file 2 — Appendix S2: ece372316‐sup‐0002‐AppendixS2.docx. [file ECE3-15-e72316-s002.docx]

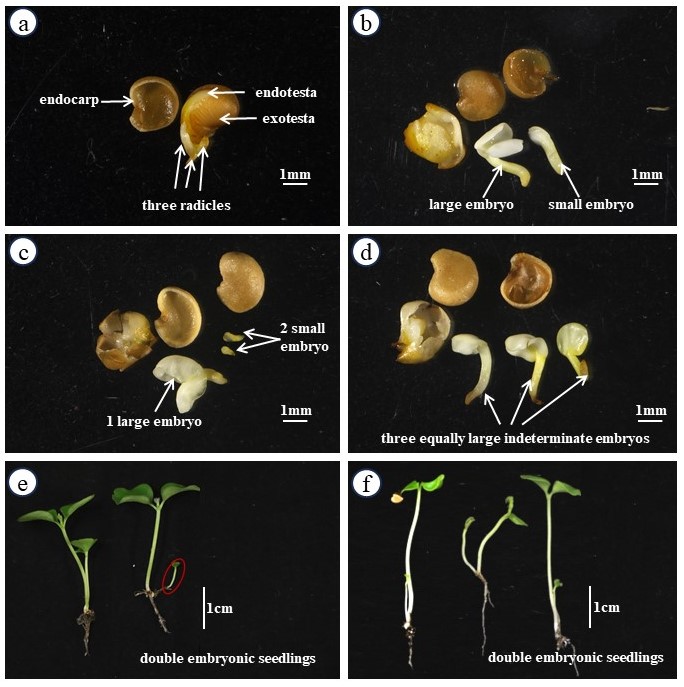


Figure S1. The development status of polyembryonic seeds of *F. gasparriniana*.

**(a)**, three embryonic seeds; **(b)**, double embryonic seeds; **(c)**, three embryonic seeds, shows one large embryo and two stunted adventitious embryos; **(d)**, three adventitious embryos of equal size; **(e)**, double embryonic seedlings with abnormal small embryo development; **(f)**, double embryonic seedlings for the seeds collected in the field.


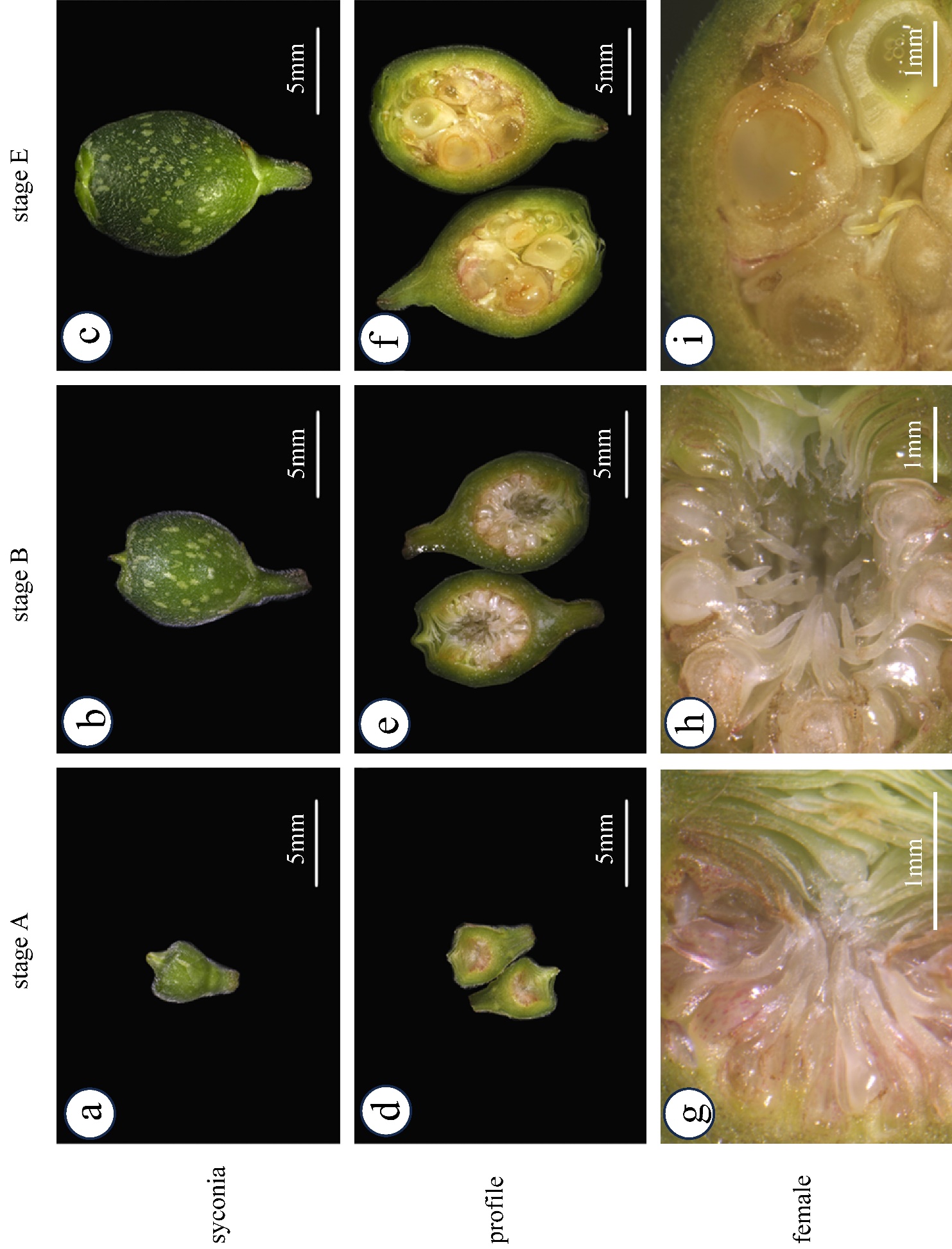


Figure S2. Different developmental stages of the female syconia of *F. gasparriniana*.


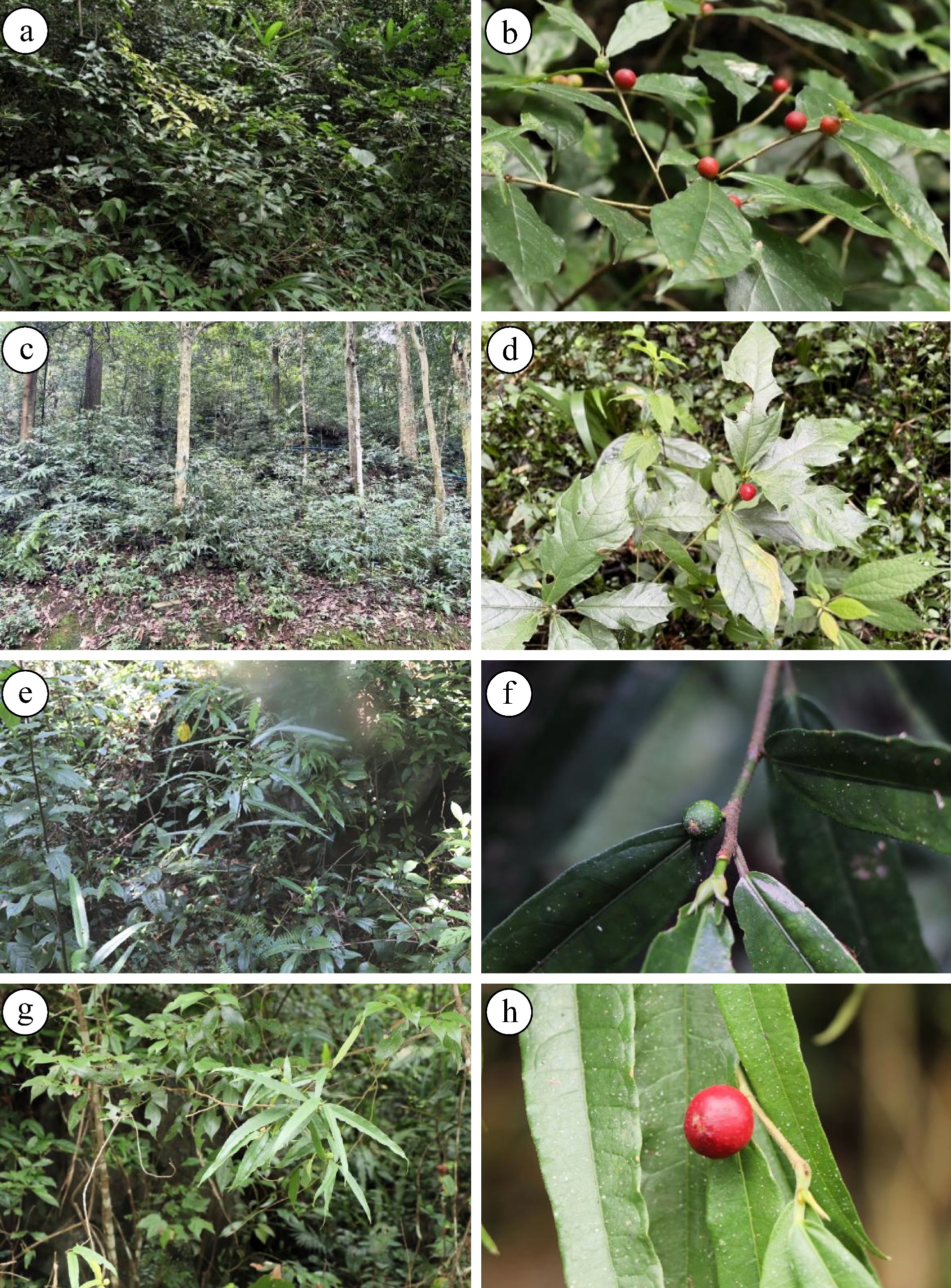


Figure S3. Natural apomictic populations of *F. gasparriniana*.

**(a, b)**, Qianling mountain; **(c, d)**, Emei mountain; **(e, f)**, Maolan; **(g, h)**, Jiayi


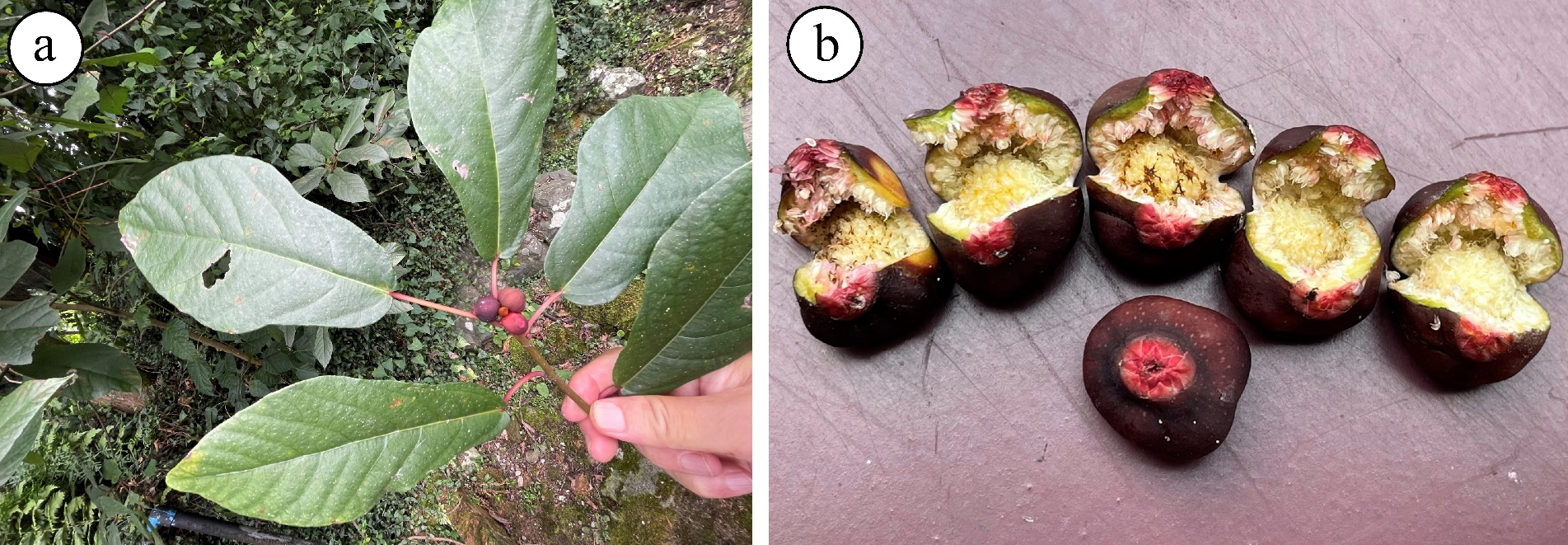


Figure S4. *F. heteromorpha* distributed sympatrically with *F. gasparriniana* in the Emei Mountain population.

**(a)**, branch; **(b)**, syconia


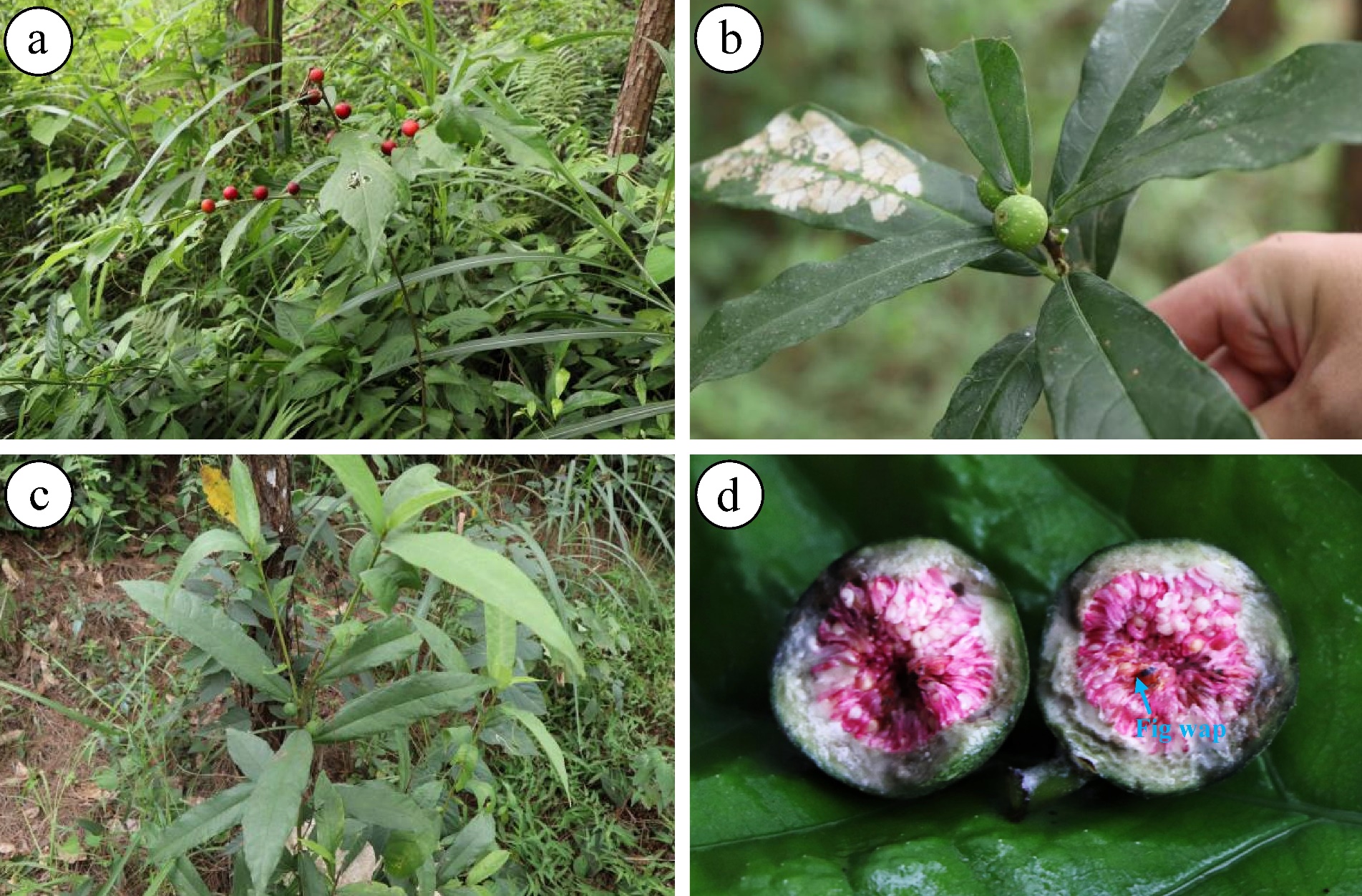


Figure S5. Natural sexually reproducing populations of *F. gasparriniana*.

**(a-c)**, plants with different traits; **(d)**, male syconia with fig wap activity


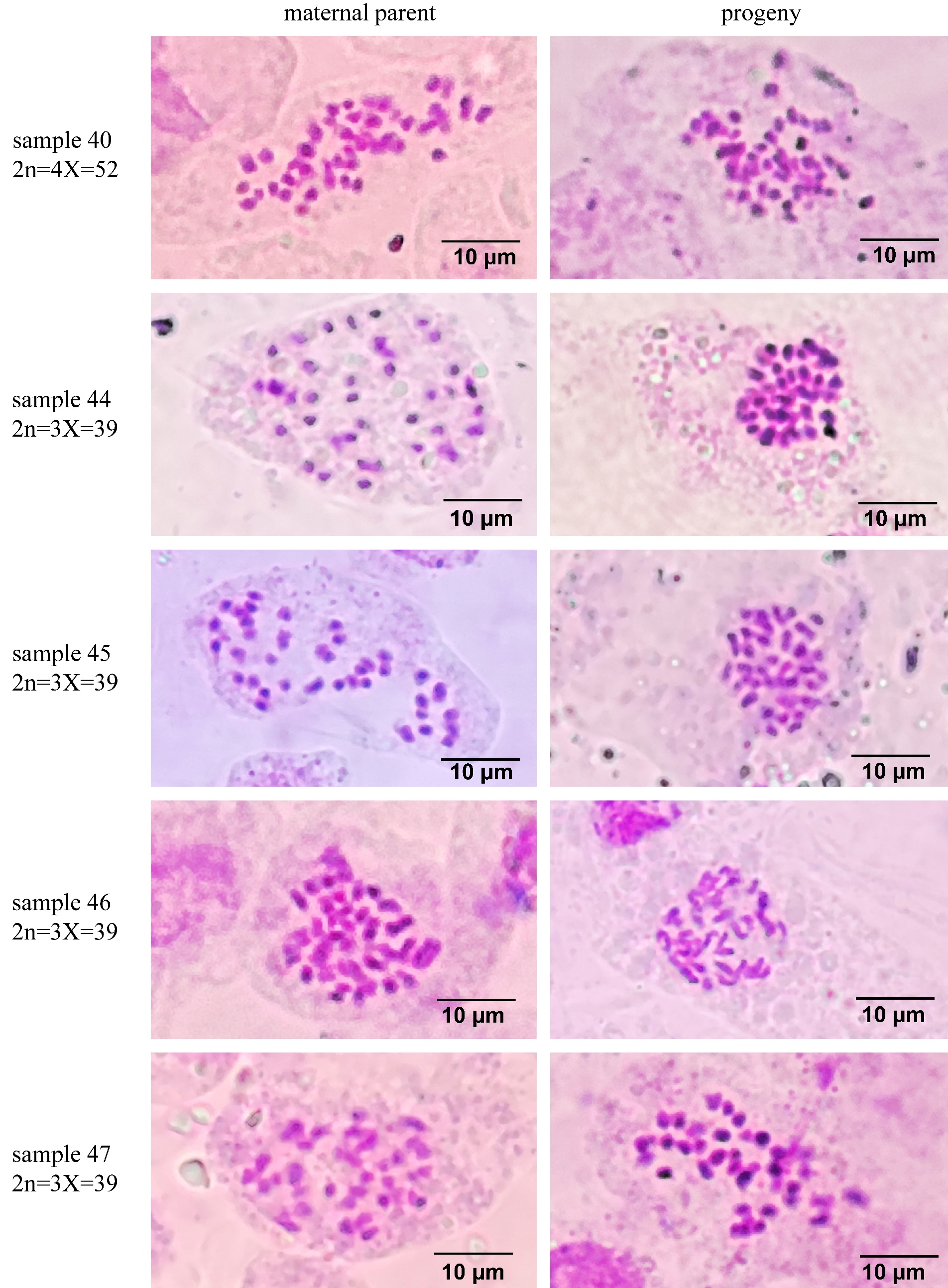


Figure S6. Chromosome diagram.


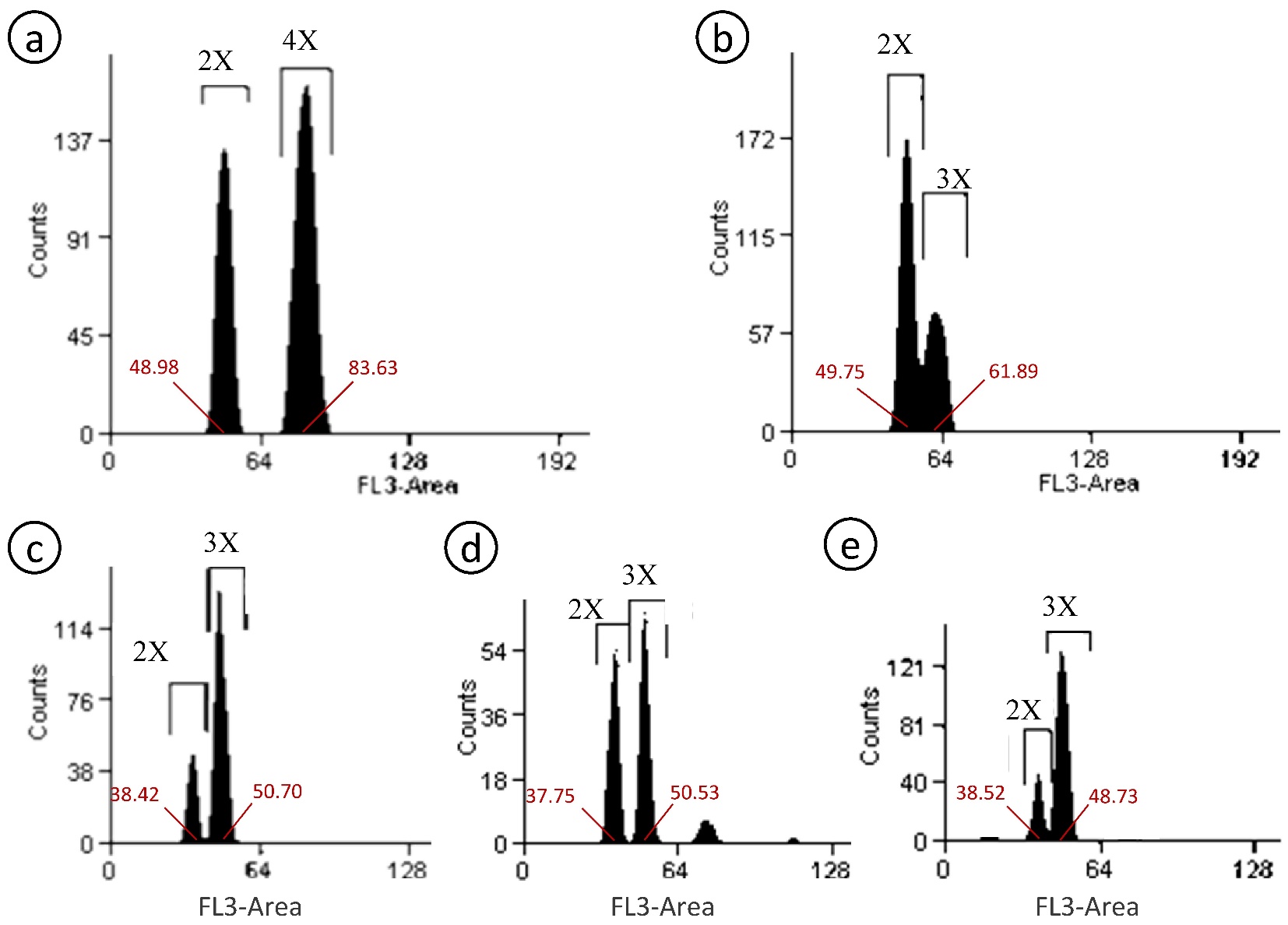


Figure S7 Flow cytometry plot of mixed-ploid samples.

**abscissa**, the fluorescence intensity of each cell nucleus (relative DNA content); **ordinate**, the number of cells; **(a)**, 40A; **(b)**, 45M; **(c)**, 26C; **(d)**, 29B; **(e)**, 29C.
